# Photoredox C–H functionalization leads the site-selective phenylalanine bioconjugation

**DOI:** 10.1038/s41598-022-23481-6

**Published:** 2022-11-08

**Authors:** Yue Weng, Chun-Jen Su, Haoyang Jiang, Chien-Wei Chiang

**Affiliations:** 1 Department of Chemistry, Soochow University, Taipei, Taiwan; 2 Ministry-of-Education Key Laboratory for the Synthesis and Application of Organic Functional Molecule &, School of Chemistry and Chemical Engineering, Hubei University, Wuhan, People’s Republic of China; 3 TLS BL23A, National Synchrotron Radiation Research Center (NSRRC), Hsinchu, Taiwan

**Keywords:** Catalysis, Chemical biology, Photochemistry

## Abstract

Site-selectively chemical bioconjugation of peptides and proteins can improve the therapeutic exploration of modified protein drugs. Only 3.8% natural abundance of phenylalanine in protein and nearly 90% of proteins contain at least one phenylalanine residue in their sequenced, showing the potential in biopharmaceutical utility of the phenylalanine bioconjugation. However, the covalent bioconjugation of native phenylalanine is one of the most challenging problems in protein modification. Herein, an approach to protein modification is described that relies on a photoredox method for the site-selective bioconjugation of phenylalanine. This methodology has been validated on peptides as well as protein insulin using a straightforward and mild condition. In addition, based on characterization by near-UV CD spectroscopy and small angle X-ray scattering (SAXS), this pyrazole labeling approach permitted the insulin hexamer to completely dissociate into the monomeric form, thus making it a potential candidate for use as rapid-acting insulin for the treatment of diabetes.

## Introduction

**Figure 1:**
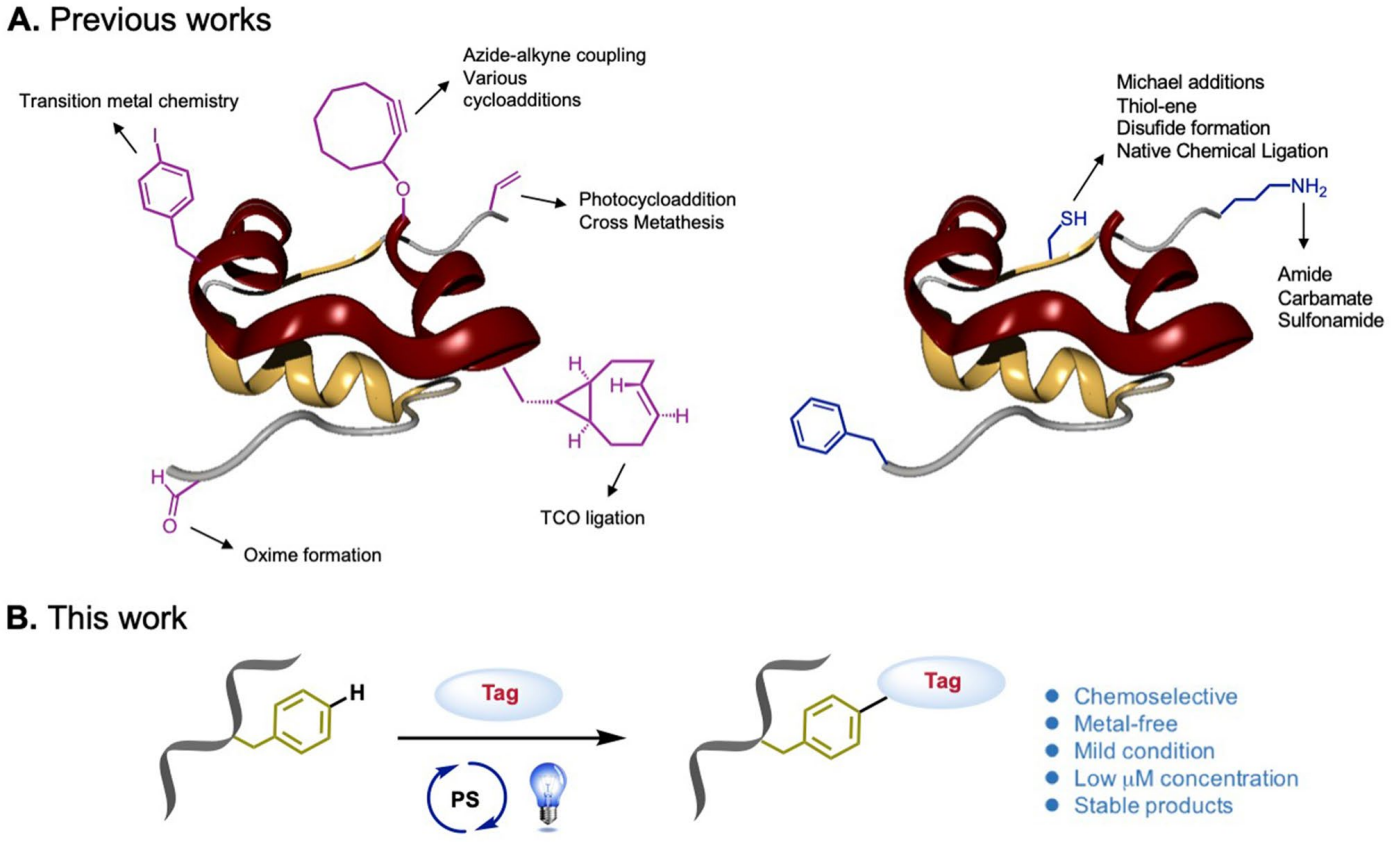
Site-selectivity approaches to protein modification. (**A**) Commonly used bioconjugal handles: non-natural handles, such as iodobenzyl, cyclopentaynyl, alkenyl, and oximyl groups, usually used for the functionalization of biomolecules; natural handles, Cys and Lys exploit their native chemical functionality. (**B**) General strategy for visible-light-induced site-selective phenylalanine bioconjugation.

Early conjugation technologies depended on the random bioconjugation of amino acid side chains in proteins, resulting in heterogeneous mixtures of labelled proteins^1^. In contrast, site-specific conjugation would result in a homogeneous population of proteins/peptides conjugates with improved pharmacological properties compared to randomly coupled molecules^2,3^. Although the incorporation of a non-natural functionality into biomolecules, such as aryl iodides, alkynes, alkenes, oximes and TCO ligation, exhibit exquisite chemoselectivity through their orthogonal chemical functionalities (Fig. 1A), site-selectively chemical methods for the direct attachment of “tags” to native amino acid residues of biomolecules, at present, is still a challenging and attractive chemistry.

Recently, because of their high reactivity, cysteine (Cys) and lysine (Lys) are the most common bioconjugal handles^4–10^. By utilizing various additional substrates, directly labelled Cys or Lys contained biomolecules could be achieved through the thiol-yne coupling or substitution reactions (Fig. 1A). In contrast, residues with aromatic side chains, such as histidine (His), tyrosine (Tyr), tryptophan (Trp), and phenylalanine (Phe) also provide the modification potentials with their aromatic systems^5,6^. Previously, the bioconjugation of His, Tyr and Trp were most commonly described by introducing the transition metal catalyzed cross-coupling reactions. However, phenylalanine was used to recognized as unexploited bioconjugal handle in the field of protein modification owing to its naturally high C–H bond energy of the phenyl residue^11^. On the other hand, since the low abundance in protein (3.8%) and approximately 90% of proteins contain at least one phenylalanine residue in their sequence, the bioconjugation of phenylalanine shows its potential pharmaceutical utility^12^. Traditional techniques for utilizing phenylalanine typically involve the use of pre-treated unnatural phenylalanine, such as *p*-acetophenylalanine (*p*AcF) and *p*-azidophenylalanine (*p*AzF) to confer site-specific bioorthogonal chemistry characteristics^13,14^. The exception is an *E. coli* strain engineered to produce unnatural Phe^15^. Nevertheless, preparing these pre-treated unnatural Phe molecules involve the use of strong acids or oxidants such as HSO_3_Cl or NaIO_3_^16^, and cannot be used in the bioconjugation of natural proteins, either in-vivo or in-vitro.

Notably, the successful bioconjugation of phenylalanine would be contributed a great milestone for the biopharmaceuticals. For example, the insulin hexamer is commonly used as a long-acting insulin in the treatment of diabetes, however, the dissociation of the hexamer form into monomeric forms is a slow and difficult process. Some rapid-acting and short-acting insulins, such as Lispro, Aspart, and Gluisine were developed recently based on the mutation of the B28Pro or B29Lys residues in the C-terminal of insulin^17^. However, approaches that involve mutating or modifying the Phe1 on N-terminal of insulin are still quite unusual^18,19^. According to the crystal structure of insulin, we assumed that while Phe1 of the N-terminal of insulin could be site-selectively modified, it would enhance the steric hindrance between each of the motifs of insulin and might cause the insulin hexamer to dissociate to produce a new type of rapid-acting insulin^18^. Therefore, directly labelling phenylalanine units in a protein would be desirable, which is needed to unlock the potential of phenylalanine as a fully diversifiable residue in a biomolecule.

In order to achieve the site-selective bioconjugation of phenylalanine, we suggested that strategies involving the use of transition metal catalysis and photoredox catalysis would allow the direct functionalization of phenylalanine^20–23^. Particularly, the procedure of photoredox catalysis is clean and simple and involves mild conditions, for example, an important contribution on photo-redox protein modification that recently reached^24^. Therefore, visible-light-induced process on bioconjugation might serve as a guidance for a general phenylalanine labelling strategy. Herein, we attempted to use a suitable photosensitizer to construct a C–N bond between a phenylalanine residue and a pyrazole derivative (Fig. 1B). The approach of photocatalysis would permit the generation of a covalent bond on the phenylalanine unit, thus avoiding instability issues in living systems. In addition, it might not only combine the introduction of chemical ‘tags’ into an inactive amino acid phenylalanine but would also authorize the position of the chemoselective modification to be controlled under mild conditions.

## Results

**Figure 2:**
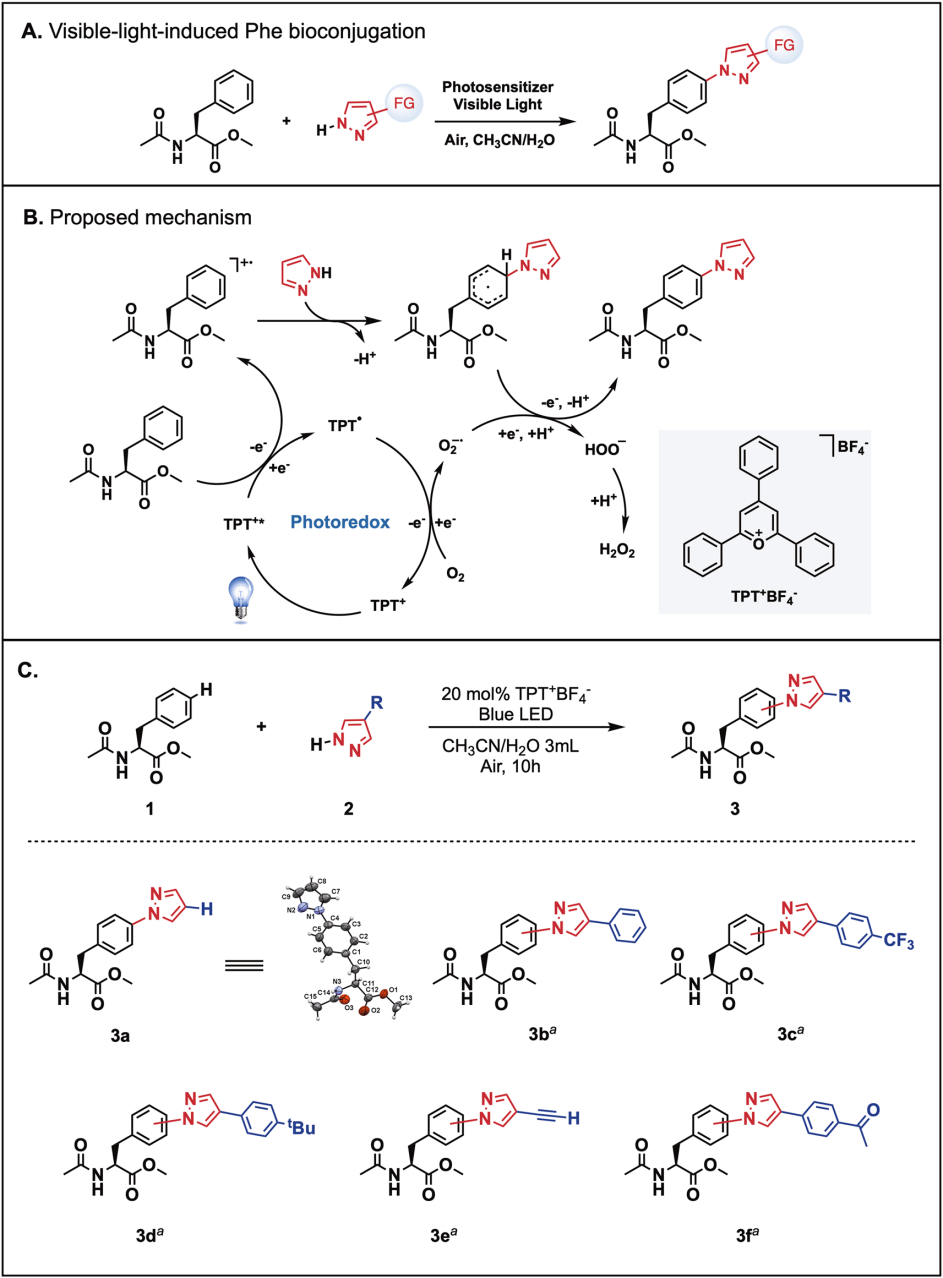
Visible-light-induced phenylalanine bioconjugation. (**A**) Reaction model of visible-light-induced phenylalanine conjugation under mild and atmospheric conditions. (**B**) The proposed mechanism for the reaction between phenylalanine and pyrazoles. (**C**) The molecular structure of **3a** was determined by X-ray crystallography. Conversion of the starting Phe **1** into the corresponding aminated products was confirmed by LC–MS. ^a^The corresponding products were detected in LC–MS with less than 10% yield.

Our approach will be based on the nucleophilic attack of pyrazole moiety to the *para*-position of an intermediate phenylalanine radical cation, which can be generated by the oxidation of methyl acetyl-l-phenylalanine by the excited state of a photosensitizer (TPT^+*^). This is followed by deprotonation and oxidization by superoxide to generate the pyrazole-bound phenylalanine product. Through the above single-electron-oxidation and deprotonation of the oxidative oxygen species, the corresponding pyrazole-bound phenylalanine would be produced (Fig. 2A,B).

Therefore, we began our study of this visible-light-induced strategy of phenylalanine bioconjugation by investigating different photosensitizers and oxidants. As a model system, by using a CH_3_CN/H_2_O (1/1, v/v) solvent system and atmospheric conditions, methyl acetyl-l-phenylalanine (**1**) was conjugated with 1.5 eq. of pyrazole (**2a**) in the presence of 20 mol% TPT^+^BF_4_^−^ under irradiation with blue LEDs, the reaction will achieve 99% conversion of phenylalanine to construct the para-substituted pyrazole-bound phenylalanine adduct 3a with 28%. However, the use of small molecular amino acids would be decomposed in the photoredox condition. Therefore, the resulting solution was also observed with the backbone decomposition fragments as the byproducts. In order to test the effect of the functional group (R) on reactivity, the further substrate scope of a series of pyrazoles (0.3 mmol, 1.5 eq.) was performed with **1** (0.2 mmol, 1.0 eq.). However, in these cases, the transformation of the desired products was poor (less than 10%) because of the strong redox potential of TPT^+^BF_4_^−^. Still, this method can be potentially used to bioconjugate phenylalanine with a variety of functionalities on the pyrazole pendant, including different electron-donating or electron-withdrawing arenes (**3b**–**3d**) as well as bioconjugation handles such as alkyne or acetyl groups (**3e** and **3f**) (Figs. 2C, S1). In addition, to get more insight into the reaction mechanism. We treated TEMPO as the radical scavenger to the model reaction solution. As a result, the desired product is only present in a trace amount, and the decomposition of amino acid was diminished, which means the reaction certainly proceeds under a radical process.

**Figure 3:**
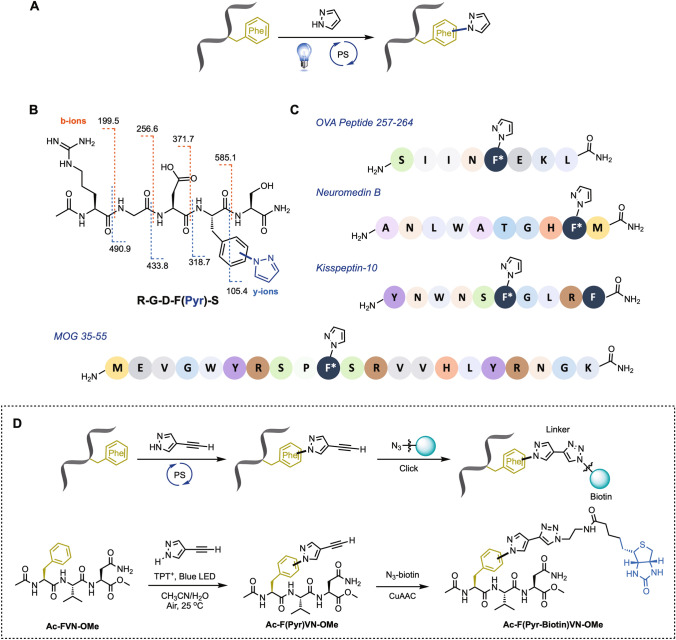
The scope of visible-light-induced strategy applied to polypeptides. (**A**) General reaction scheme. (**B**) MS/MS analysis of the fragmentation of RGDF(Pyr)S. (**C**) Substrate scope of polypeptides. All peptides are commercially available. * indicates site of pyrazole-tagged functionalization. (**D**) Two-step process for functionalizing the phenylalanine-contained tripeptide (**Ac-FVN-OMe**). The visible-light-induced bioconjugation of **Ac-FVN-OMe** with 4-ethynyl-1*H*-pyrazole and the further synthesis of the biotin tagged tripeptide **Ac-F(Pyr-Biotin)VN-OMe** by CuAAC reaction.

Importantly, with the optimized reaction conditions in hand, we next expected to examine the applicability of this photoredox approach for polypeptides with biological activity (Fig. 3A). The site-selective modification of a variety of fully unprotected peptides from 5-mers to 20-mers, such as a RGD contained peptide, Neuromedin B, Kisspeptin-10, and a part of OVA and MOG peptides, can be achieved successfully at room temperature (Fig. 3B,C). Notably, this site-selective modification can attach a pyrazole to the RGD peptides, which is the most common peptide motif responsible for cell adhesion to the extracellular matrix, to provide a diverse application in biological research. RGD peptides have found many applications in drug discovery and medical devices. For instance, the RGD-recognizing *ανβ6* integrin have been considered attractive target for the tumor treatment and diagnosis^25,26^.

A biotin-labelled insulin molecule was reported to show an increased insulin sensitivity, leading to a decrease in blood glucose levels^27^. Therefore, in order to explore the further bio-orthogonal possibility and biopharmaceutical application of this methodology. We synthesized a tripeptide (**Ac-FVN-OMe**) which serves as a mimic of a part of the N-terminal of the B chain of insulin. Subsequently, with the addition of a bio-orthogonal handle containing pyrazole, 4-ethynyl-1*H*-pyrazole, to **Ac-FVN-OMe**, the phenylalanine residue could be attached specifically and then afforded a 4-ethynyl-1*H*-pyrazole tagged tripeptide **Ac-F(Pyr)VN-OMe**. Through a copper-catalyzed azide-alkyne cycloaddition (CuAAC) reaction by introducing a N_3_-biotin to **Ac-F(Pyr)VN-OMe**, afterwards, biotin can be chemoselectively tagged to **Ac-FVN-OMe** (Fig. 3D)^28^ and demonstrated the advance bio-orthogonal opportunity of the photoredox approach. The merging of the site-specific incorporation of a small bio-orthogonal functional group into proteins and polypeptides with bio-orthogonal chemistry has created exciting opportunities for extending the power of organic reactions to living systems.

With a direct and efficient route to the photocatalytic pyrazolization of phenylalanine-contained biomolecules being established, their compatibility with protein bioconjugation should be investigated in more detail. To test the capability of this photoredox procedure in this context, a native mammalian protein insulin, which contains one free phenylalanine (B1Phe) in a total of 51 amino acid residues (m/z = 5777 Da) will be exploited. Insulin is composed of two main chains linked by two disulfide bridges, because the Phe1 residue appears to be exposed on the surface of the insulin in the crystal structure, we expect that the treatment of a pyrazole with this photoredox protocol would result in the binding of the pyrazole to insulin. Consequently, we verified this visible-light-induced protocol on insulin. After the characterizations of LC-HRMS, MALDI-TOF-MS and MSMS spectroscopies (see Supplementary Information), it shows that the photocatalytic pyrazole-conjugated reaction was site-selectively complete within 2 h, as evidenced by the fact that the apparent peak for insulin (m/z = 5777 Da) was shifted to a peak corresponding to a pyrazole-bound insulin (m/z = 5844 Da).

**Figure 4:**
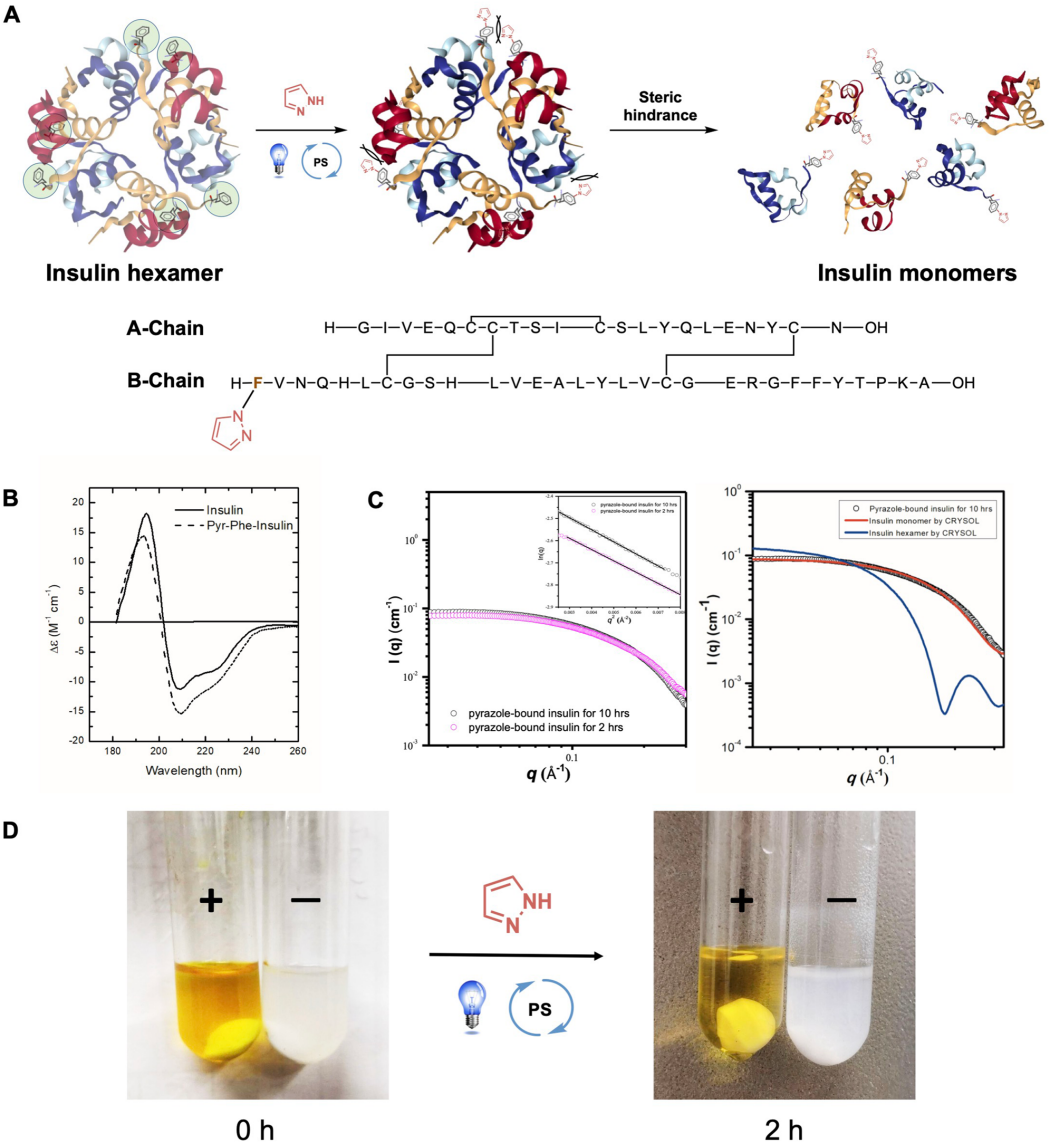
Visible-light-induced pyrazole tagging for the synthesis of phenylalanine-targeted insulin conjugates and its activity. (**A**) Site-selective visible-light-induced pyrazole tagging strategy. Crystal structure of Insulin (PDB: 4ins) with six native phenylalanine residues shown as green circles. (**B**) Near-UV circular dichroism of insulin (—) and Pyr-tagged insulin (–). For circular dichroism studies, all samples were measured at identical protein concentrations using the same 100-μm-pathlength cuvette. (**C**) Left: Representative SAXS data obtained for pyrazole-tagged insulin as a function of reaction time. Inset shows the Guinier curve-fitted lines for Rg values to the experimental data; Right: The small angle X-ray scattering studied of VPT conjugated insulin (black circle). The data is fitted using the program CRYSOL (solid curves). The solid lines represent the theoretical curves calculated from the crystal structures of the insulin monomer (PDB: 2jv1, red line) and the insulin hexamer (PDB: 1ai, blue line) (**D**) Insulin, when formulated with pyrazole conjugates, shows soluble after the reaction of 2 h, whereas it readily precipitates from solution when dissolved alone. Shown is an illustrative example of this effect following pyrazole-tagged insulin (+) compared with insulin alone in solution (−).

As we mentioned before, insulin ordinarily exists as a hexamer form, and the dissociation of the hexamer into monomeric insulin is a quite slow progression. However, we suspected when the Phe1 of insulin is attached by a pyrazole, the insulin hexamer would rapidly become dissociated into monomers because of the steric hindrance between each domain (Fig. 4A). In order to prove this concept, the tertiary structure of pyrazole-tagged insulin and original insulin have been studied and compared by near-UV CD spectroscopy, where the original insulin shows two negative bands of almost equal magnitude at 210 and 220 nm, and a positive maximum at 196 nm, and pyrazole-bound protein displays a slightly change toward the spectrum of original insulin (Fig. 4B). In comparison to the near-UV CD spectroscopy of monomeric mutated insulin in the literature, the above results indicated that the pyrazole-tagged insulin should show to be monomeric, but retaining native-like tertiary structure.

Meanwhile, we investigated the real motif of the pyrazole-tagged insulin by small-angle x-ray scattering (SAXS). Particularly, SAXS is powerfully used to obtain shape information and dimensional parameters of biological macromolecules. Figure 4C illustrates the experimental SAXS data and a Guinier plot displayed in the inset. In order to confirm that the sample was monodispersity, we investigated the Guinier approximation for its validity in the region of the intensity curve^29,30^. The linearity of this region confirmed its monodispersity. The calculated radius of gyration (Rg) values for the pyrazole-bound insulin for 10 h and the pyrazole-bound insulin for 2 h in solution were 12.77 ± 0.5 Å and 12.36 ± 0.6 Å, respectively, obtained using the Guinier approximation. These Rg values for pyrazole-bound insulin are consistent with that for the human insulin monomer (12.0 ± 0.5 Å)^31^. By utilizing SAXS to study the bioconjugated insulin in solution, we found that the pyrazole-tagged insulin was separated from the insulin hexamer as an insulin monomer. In comparison to the theoretical scattering curves calculated for the crystal structures of the monomeric and hexameric forms of human insulin (solid lines), the experimental data were nearly overlapped with the simulated curve of human insulin monomer, indicating the pyrazole-modified insulin in solution is a monomeric form (Fig. 4C, right).

Poor insulin hexamer stability has proved limiting to its use in diabetes therapy. When insulin was formulated with pyrazole by using the photoredox method, the pyrazole-tagged insulin showed soluble in solution, whereas insulin alone precipitated from solution under 2 h (Fig. 4D). Moreover, the pyrazole-bound insulin monomer is stable under ambient conditions, and can be stored in a closed vial under air at 4 °C for over one month. With the above benefits, this pyrazole-conjugated insulin is worthy of being investigated for further applications and for the treatment of diabetes. Interestingly, as shown in Figs. S11 and 4C, larger biomolecules such as insulin, instead of single amino acids, could observe modified insulin converted well and transformed its hexamer form to monomers. This result demonstrates two viewpoints. First, the larger molecules exhibit a higher tolerance in the photoredox condition because the environment might contain some ions and are more rigid, which will provide some protection to the reaction site. Second, although the functionalization of a single amino acid pattern of Phe is poor due to its self-unitability, this observation still can provide further applications in enormous peptide/protein modification.

In conclusion, a simple, mild, and rapid method for the preparation of pyrazole-bound phenylalanine from the parent phenylalanine using visible-light-induced tagging strategy is reported here. This strategy has been successfully applied to the site-specific bioconjugation of insulin, which permits the insulin hexamer to spontaneously dissociate into the monomeric form^32^. We anticipate that advances in visible-light-induced phenylalanine bioconjugation will address a number of current limitations and continue to enlist both biomolecule and small-molecule chemists, leading to an expanding library of interdisciplinary methods.

## Methods

### General considerations

Unless otherwise stated, analytical grade solvents and commercially available reagents were used without further purification. Amino acids were bought at Energy chemistry; biotin, phenylboronic acid, pyrazole and 4-I-1-H-pyrazole was purchased from Bide Pharmatech; porcine insulin was bought at Aladdin. Pyrazoles were generally prepared according to literature procedures^1,2^. All manipulations were carried out by using standard Schlenk techniques. Thin layer chromatography (TLC) employed glass 0.25 mm silica gel plates. Flash chromatography columns were packed with 200–300 mesh silica gel in hexane/ethyl acetate. Gradient flash chromatography was conducted eluting with a continuous gradient from petroleum ether to dichloromethane.

^1^H-, ^13^C-, and ^19^F-NMR spectra were recorded on a Bruker 400 MHz NMR spectrometer. For ^1^H-NMR, chemical shifts (δ) were given in ppm relatives to internal standard (TMS at 0 ppm, CDCl_3_ at 7.26 ppm, DMSO-d_6_ at 49.00 ppm). For ^13^C-NMR, chemical shifts (δ) were reported in ppm using solvent as internal standard (CDCl_3_ at 77.16 ppm, MeOH-d_4_ at 49.00 ppm, Acetone-d_6_ at 206.26 ppm). Data are reported as: s = singlet, d = doublet, t = triplet, q = quartet, p = pentet, m = multiplet, br = broad; coupling constants in Hz; integration.

High resolution mass spectra (HRMS) were obtained by use of a Bruker Compact TOF mass spectrometer in electrospray ionization mode (ESI+). All of the MALDI-TOF-MS and MALDI-TOF-MS/MS spectra were acquired using 5800 MALDI-MS (AB SCIEX, Concord, Canada) equipped with a 355 nm Nd: YAG laser in the reflector positive mode. Samples of 0.6 μL mixed with 0.6 μL freshly prepared CHCA matrix were directly loaded onto the stainless steel MALDI plate and allowed to dry in a gentle stream of warm air. Samples were ablated with a power of 3500 while the laser rastered over the target surface. A total of 2000 laser shots were employed in each sample spot. The MS and MS/MS data processing was further performed by DataExplorer 4.0 (AB SCIEX, Concord, Canada).

### General procedure for bioconjugation of phenylalanine and pyrazoles

To a solution of methyl acetyl-l-phenylalaninate **1** (0.2 mmol, 1 equiv., 44.2 mg) in CH_3_CN/H_2_O (1.5 mL/1.5 mL) was added pyrazoles **2** (0.3 mmol, 1.5 equiv.) in presence of photosensitizer TPT^+^BF_4_^−^ (0.04 mmol, 20 mol%, 15.8 mg) under air atmosphere and irradiated by 3 W blue LEDs at 25 °C for 10 h. After completion of the reaction, the solvents were removed under reduced pressure by rotary evaporation to give a residue, which was then purified by column chromatography on silica gel (hexane: ethyl acetate mixtures to afford the product **3**.

### Purification and characterization of methyl (S)-3-(4-(1H-pyrazol-1-yl)phenyl)-2-acetamidopropanoate (**3a**)

Methyl acetyl-l-phenylalanine **1** was reacted according to the above procedure with pyrazole **2a** (0.3 mmol, 20.4 mg), and TPT^+^BF_4_^−^. The crude reaction mixture was plugged through silica gel and concentrated. Suitable crystals of **3a** for structure determination were obtained by crystallized from CH_2_Cl_2_/hexane in 28% yield. ^1^H-NMR (400 MHz, CDCl_3_) δ 7.90 (d, J = 2.2 Hz, 1H), 7.72 (d, J = 1.6 Hz, 1H), 7.63 (d, J = 8.5 Hz, 2H), 7.18 (d, J = 8.5 Hz, 2H), 6.47 (dd, J = 2.2, 1.6 Hz, 1H), 4.93–4.89 (m, 1H), 3.74 (s, 3H), 3.23–3.11 (m, 2H), 2.01 (s, 3H) ppm. ^13^C-NMR (101 MHz, CDCl_3_) δ 172.07, 169.82, 141.20, 139.30, 134.25, 130.35, 126.79, 119.37, 107.77, 53.21, 52.55, 37.37, 23.23 ppm. (Fig. S15) m/z HRMS(ESI) found [M+Na]^+^ 310.1170, C_15_H_17_N_3_O_3_Na^+^ requires 310.1162 (Fig. S1A).

### Characterization of (S)-3-(4-(1H-pyrazol-1-yls)phenyl)-2-acetamidopropanoate (**3b**–**3f**)

*(S)-3-(4-(1H-4-phenyl-1H-pyrazol-1-yl)phenyl)-2-acetamidopropanoate (****3b****)*: m/z HRMS(ESI) found [M+Na]^+^ 386.1464, C_21_H_21_N_3_O_3_Na^+^ requires 386.1475.

*(S)-3-(4-(1H-4-(4-(trifluoromethyl)phenyl)-1H-pyrazol-1-yl)phenyl)-2-acetamidopropanoate (****3c****)*: m/z HRMS(ESI) found [M+Na]^+^ 454.1360, C_22_H_20_F_3_N_3_O_3_Na^+^ requires 454.1349.

*(S)-3-(4-(1H-4-(4-(tert-butyl)phenyl)-1H-pyrazol-1-yl)phenyl)-2-acetamidopropanoate (****3d****)*: m/z HRMS(ESI) found [M+H]^+^ 422.2078, C_25_H_30_N_3_O_3_^+^ requires 420.2282.

*(S)-3-(4-(1H-4-(4-(ethynyl-1H-pyrazol-1-yl)phenyl)-2-acetamidopropanoate (****3e****)*: m/z HRMS(ESI) found [M+Na]^+^ 334.1161, C_17_H_17_N_3_O_3_Na^+^ requires 334.1162.

*(S)-3-(4-(1H-4-(4-(1-(4-(1H-pyrazol-4-yl)phenyl)ethanonyl)phenyl)-2-acetamidopropanoate (****3f****)* : m/z MS(ESI) found [M+H]^+^ 405.1000, C_23_H_23_N_3_O_4_^+^ requires 405.1680.

## Supplementary Information

Supplementary Information.

## Data Availability

The datasets generated and analysed during the current study are available in the Crystallography Open Database (COD) repository, https://www.crystallography.net/cod/3000406.html.
