# Supplementary material for: Photoredox C–H functionalization leads the site-selective phenylalanine bioconjugation
Source: Sci Rep. 2022 Nov 8;12:18994. doi: 10.1038/s41598-022-23481-6 (PMC9643349; doi:10.1038/s41598-022-23481-6)
Supplement: Supplementary file 1 — Supplementary Information. [file 41598_2022_23481_MOESM1_ESM.docx]

Supplementary Materials for

**Photoredox C-H Functionalization Leads the Site-selective Phenylalanine Bioconjugation**

Yue Weng^2^; Chun-Jen Su^3^; Haoyang Jiang^2^; Chien-Wei Chiang^1*^

correspondence to: cwchiang@scu.edu.tw (C.-W. C.)

**This PDF file includes:**

Materials and Methods

Figs. S1 to S15

Tables S1 to S2

References

General Considerations

Supplementary Methods

Table S1 Investigation of photosensitizers

Table S2. Control experiments

1. Chemical synthesis of photosensitizer TPT^+^BF_4_^-^
2. General procedure of protected amino acid
3. Chemical synthesis procedure of tripeptide Ac-FVN-OMe
4. Chemical synthesis of pyrazoles
5. Synthesis of biotin-azide
6. General procedure for bioconjugation of phenylalanine contained tripeptide Ac-FVN-OMe
7. Synthesis of biotin tagged tripeptide Ac-F(Pyr-Biotin)VN-OMe by CuAAC reaction.
8. Procedure for visible-light-induced pyrazole tagging conjugated phenylalanine-targeted biomolecules
9. SAXS measurements

Supplementary Figures and Data

S1. LC-MS analysis of amino acid modifications.

S2. Visible-light-induced reactivity of pyrazole with other natural amino acids.

S3. ESI-HRMS analysis of tripeptide modification.

S4. ESI-HRMS analysis of the bio-orthogonal product of the tripeptide Ac-FVN-OMe

S5 – S9 MALDI-TOF-MS/MS data of modified polypeptides

S10. MALDI-TOF-MS/MS data of modified insulin

S11. LC-MS analysis of insulin and modified insulin.

S12. Control experiments of visible-light-induced pyrazole tagging on insulin.

S13. X-Ray crystal data for pyrazole-phenylalanine adduct 3a

S14 – S15 NMR data of synthetic compounds

References and Notes

Table S1. Investigation of photosensitizers^a^

^a^Conditions: a CH_3_CN/H_2_O (1.5 mL/1.5 mL) solution of **1**, **2a** and **PS** was irradiated by blue LED for 10 h in the atmosphere. ^b^Conversions and yields were determined by FID-GC, using biphenyl as the internal standard.

Table S2. Control experiments

^a^Standard conditions: a CH_3_CN/H_2_O (1.5 mL/1.5 mL) solution of **1**, **2a** and **PS** was irradiated by blue LED for 10 h in the atmosphere. ^b^Conversions and yields were determined by FID-GC, using biphenyl as the internal standard.

**1. Chemical synthesis of photosensitizer TPT^+^BF_4_^-^**

According to the literature procedure,(*3*) BF_3_•OEt_2_ is slowly added to a mixture of the carbonyl compounds (if starting materials are solids, they are dissolved in a small amount of toluene). After heating for 2 h at 100 ºC the thus formed diethyl ether is evaporated and the oily product dissolved in acetone. Upon the addition of diethyl ether the product precipitates. Purfication is performed by multiple recrystallization from acetone.

**2. General procedure of protected amino acid**

In an oven-dried round-bottom flask (100mL) amino acid (20 mmol) was dissolved in anhydrous MeOH (40 mL). Stirred, cooled to 0 °C. Carefully added (2.8 mL, 40 mmol) SOCl_2_ to the solution. The reaction mixture was then warmed to room temperature and stirred overnight. The solvent was removed by rotary evaporation to afford amino ester hydrochloride residue. Without further purification, the residue and triethyl amine (5.6 mL, 40 mmol) was added in anhydrous DCM (40 mL) and stirred for 15 min at 0 °C. Acetyl chloride (1.4 mL, 20 mmol) was added to the reaction solution dropwisely. Stirring was continued for 12 h while allowing the mixture to warm up to r.t.. The reaction was washed with saturated NaHCO_3_ solutions (50 mL×2) and 10% HCl (50 mL×1) to remove any unreacted starting material. The combined organic extracts were dried (MgSO_4_), filtered, and concentrated in vacuo. Purification of the residue by flash chromatography (SiO_2_, hexane:ethyl acetate mixtures) led to the desired protected amino acid.

**methyl acetyl-L-phenylalaninate (1)**

^1^H NMR (400 MHz, DMSO-*d*_6_) δ 8.38 (d, *J* = 7.7 Hz, 1H), 7.35 – 7.15 (m, 5H), 4.45 (m, 1H), 3.42 (s, 3H), 3.01 (dd, *J* = 13.7, 5.6 Hz, 1H), 2.87 (dd, *J* = 13.7, 9.4 Hz, 1H), 1.79 (s, 3H) ppm. ^13^C NMR (101 MHz, DMSO-*d*_6_) δ 171.28 (d, *J* = 283.9 Hz), 137.71, 129.47, 128.71, 127.01, 54.11, 52.26, 37.18, 22.68 ppm. (Figure S16)

**methyl acetyl-L-tyrosinate**

^1^H NMR (400 MHz, DMSO-*d*_6_) δ 9.27 (s, 1H), 8.30 (d, *J* = 7.7 Hz, 1H), 6.99 (d, *J* = 8.4 Hz, 2H), 6.66 (d, *J* = 8.5 Hz, 2H), 4.38 – 4.30 (m, 1H), 3.57 (s, 3H), 2.87 (dd, *J* = 13.8, 5.8 Hz, 1H), 2.74 (dd, *J* = 13.8, 9.1 Hz, 1H), 1.79 (s, 3H) ppm. ^13^C NMR (101 MHz, DMSO-*d*_6_) δ 172.83, 169.84, 156.42, 130.41, 127.67, 115.49, 54.47, 52.20, 36.49, 22.70 ppm. (Figure S17)

**methyl acetyl-L-tryptophanate**

^1^H NMR (400 MHz, DMSO-*d*_6_) δ 10.88 (s, 1H), 8.35 (d, *J* = 7.5 Hz, 1H), 7.49 (d, *J* = 7.8 Hz, 1H), 7.35 (d, *J* = 8.1 Hz, 1H), 7.16 (s, 1H), 7.08 (t, *J* = 8.0 Hz, 1H), 7.00 (t, *J* = 7.8 Hz, 1H), 4.50 (td, *J* = 8.1, 5.8 Hz, 1H), 3.58 (s, 3H), 3.15 (dd, *J* = 14.6, 5.7 Hz, 1H), 3.03 (dd, *J* = 14.5, 8.5 Hz, 1H), 1.82 (s, 3H) ppm. ^13^C NMR (101 MHz, DMSO-*d*_6_) δ 173.05, 169.95, 136.55, 127.50, 124.12, 121.47, 118.92, 118.43, 111.93, 109.95, 53.64, 52.26, 27.56, 22.76 ppm. (Figure S18)

**methyl acetyl-L-histidinate**

^1^H NMR (400 MHz, DMSO-*d*_6_) δ 8.31 (s, 1H), 7.55 (s, 1H), 6.82 (s, 1H), 4.46 (m, 1H), 3.60 (s, 3H), 2.88 (m, 2H), 1.83 (s, 3H) ppm. ^13^C NMR (101 MHz, DMSO-*d*_6_) δ 172.74, 169.78, 135.38, 133.68, 116.89, 52.93, 52.21, 29.50, 22.78 ppm. (Figure S19)

**methyl acetyl-L-cysteinate**

^1^H NMR (400 MHz, Chloroform-*d*) δ 6.42 (s, 1H), 4.89 (m, 1H), 3.79 (s, 3H), 3.01 (m, 2H), 2.06 (s, 3H), 1.34 (t, *J* = 9.0 Hz, 1H) ppm. ^13^C NMR (101 MHz, CDCl_3_) δ 170.59, 169.79, 53.49, 52.82, 26.85, 23.15 ppm. (Figure S20)

**methyl acetyl-L-methioninate**

^1^H NMR (400 MHz, Chloroform-*d*) δ 6.68 (d, *J* = 8.0 Hz, 1H), 4.62 (m, 1H), 3.67 (s, 3H), 2.44 (t, *J* = 7.5 Hz, 2H), 2.06 (m, 1H), 2.01 (s, 3H), 1.95 (s, 3H), 1.89 (m, 1H) ppm. ^13^C NMR (101 MHz, Chloroform-*d*) δ 172.45, 170.03, 52.26, 51.26, 31.31, 29.74, 22.78, 15.18 ppm. (Figure S21)

**methyl acetyl-L-cystinate**

^1^H NMR (400 MHz, Chloroform-*d*) δ 6.71 (d, *J* = 7.6 Hz, 1H), 4.84 (m, 1H), 3.74 (s, 3H), 3.16 (m, 2H), 2.03 (s, 3H). ^13^C NMR (101 MHz, CDCl_3_) δ 170.85, 170.11, 52.70, 51.60, 40.51, 22.97. (Figure S22)

**3. Chemical synthesis procedure of tripeptide Ac-FVN-OMe**

Step1

.

A solution of the N-Fomc-L-asn(N-trityl)-OH (10 mmol) in MeOH was stirred at 0 °C for 10 min and continuously with SOCl_2_ for 5 min. Stirring was continued for 12 h while allowing the mixture to warm up to RT. The reaction mixture was concentrated in vacuo. Purification of the residue by flash chromatography (SiO_2_, hexane:ethyl acetate mixtures) to afford N-Fomc-L-asn(N-trityl)-OMe.

Step2

Piperidine (5 mL) was added to a solution of N-Fomc-L-asn(N-trityl)-OMe (9 mmol) in CH_3_CN (50 mL), and the resulting mixture was stirred at 25 °C for 2 h to ensure complete removal of the Fmoc protecting group. After concentration in vacuo, the residuewas purified by flash chromatography (SiO_2_, hexane:ethyl acetate mixtures).

Step3


Acetic anhydride (12 mmol) was added continuously in 5 min to a solution of phenylalanine (10 mmol) in saturated NaHCO_3_ solution. After stirring overnight, the reaction mixture was extracted by CH_2_Cl_2_, and washed with 10% HCl (50 mL×1) and water (50 mL×1) to remove any unreacted starting material. The combined organic extracts were dried (MgSO_4_), filtered, and concentrated in vacuo without further purification.

Step4

A solution of the valine (10 mmol) in MeOH was stirred at 0 °C for 10 min and continuously with SOCl_2_ for 5 min. Stirring was continued for 12 h while allowing the mixture to warm up to RT. The reaction mixture was concentrated in vacuo without further purification.

Step5

MeO-Val-NH_3_^+^Cl^-^ (9 mmol) was dissolved in DMF (40 mL). In another flask, a solution of Ac-Phe-OH (9 mmol) in DMF (40 mL) was treated with HOBt (10 mmol) and HBTU (10 mmol). After 10 min, this mixture and Et_3_N (21 mmol) were sequentially added to the above free amino ester. The reaction was stirred at 25 °C for 8 h. After regular workup, the resulting crude product was purified by flash chromatography (EtOAc/ hexanes) to afford linear dipeptide compound.

Step6

A solution of Ac -protected dipeptide ester（8 mmol） and LiOH（16 mmol） in 2:10:1 MeOH: THF: H_2_O（50 mL） was stirred under cooling at room temperature overnight. The reaction solution was cooled to 0°C and acidified with 2 M HCl and then extracted with CH_2_Cl_2_ (50 mL ×2). The combined extracts were washed with brine (50 mL ×1), dried over anhydrous Na_2_SO_4_ and concentrated *in vacuo* to give the hydrolyzed product as white solid.

Step7

H_2_N-asn(N-trityl)-OMe (5 mmol) was dissolved in DMF (20 mL). In another flask, a solution of Ac-Phe-Val-OH (5 mmol) in DMF (20 mL) was treated with HOBt (6 mmol) and HBTU (6 mmol). After 10 min, this mixture and Et_3_N (11 mmol) were sequentially added to the above free amino ester. The reaction was stirred at 25 °C for 8 h. After regular workup, the resulting crude product was purified by flash chromatography (EtOAc/ hexanes) to afford Ac-Phe-Val-Asn(N-trityl)-OMe.

Step8

Ac-Phe-Val-Asn(N-trityl)-OMe(2 mmoL) was suspended in anhydrous DCM (5 mL), and TFA(5 ml) was dropwise added. String was continued for 2 h. The reaction mixture was concentrated in vacuo, and purified by flash chromatography (EtOAc/ hexanes) to afford Ac-Phe-Val-Asn-OMe (**Ac-FVN-OMe**).

^1^H NMR (400 MHz, DMSO-*d*_6_) δ 8.34 (d, *J* = 7.4 Hz, 1H), 8.10 (d, *J* = 8.4 Hz, 1H), 7.90 (d, *J* = 9.0 Hz, 1H), 7.43 (s, 1H), 7.25 (d, *J* = 4.3 Hz, 4H), 7.21 – 7.13 (m, 1H), 6.97 (s, 1H), 4.57 (q, *J* = 6.4 Hz, 2H), 4.21 (dd, *J* = 8.7, 6.6 Hz, 1H), 3.57 (s, 3H), 2.99 (dd, *J* = 13.9, 3.8 Hz, 1H), 2.71 (dd, *J* = 13.8, 10.4 Hz, 1H), 2.58 (dd, *J* = 15.8, 5.9 Hz, 1H), 2.46 (d, *J* = 6.8 Hz, 1H), 1.97 (tt, *J* = 13.2, 5.8 Hz, 1H), 1.74 (s, 3H), 0.84 (dd, *J* = 9.8, 6.9 Hz, 6H) ppm. ^13^C NMR (101 MHz, DMSO-*d*_6_) δ 172.15, 171.80, 171.25, 171.18, 169.67, 138.54, 129.61, 128.46, 126.64, 57.58, 54.25, 52.31, 37.71, 36.88, 31.35, 22.88, 19.50, 18.31 ppm. (Figure S23)

**4. General procedure of pyrazoles**

Step 1

Trityl chloride (1.58g, 5.67mmol) was added to a stirred cold (0-5°C) solution of 4-iodo pyrazole (1 g, 5.15mmol) and triethylamine (1.04g, 10.3mmol) in DCM (12mL). Stirring was continued at room temperature overnight. Cold water was then added and the product was extracted with DCM and the organic layer was washed with saturated sodium bicarbonate solution followed by brine. The organic phase collected was dried over Na_2_SO_4_ and concentrated under reduced pressure. The residue was purified by column chromatography (using neutral alumina and 2% EtOAc in hexane as eluent) to afford 1.9g (84.4% yield) of 4-iodo-1-trityl-1H-pyrazole.

Step 2

Na_2_CO_3_ (727 mg, 6.86mmol) was added to a stirred solution of 4-iodo-1- trityl-pyrazole (1.5g, 3.43mmol) in THF:H_2_O (1:1, 20mL). Pd(PPh_3_)_4_ (790mg, 0.686mmol) and aryl-boronic acid (6.86mmol) were then added and the reaction mixture was heated to reflux for 2 hrs. The reaction mixture was then diluted with water and the product was extracted with ethyl acetate. The organic layer was washed with saturated brine solution, dried over sodium sulfate and concentrated to in vacuo. Purification by column chromatography (using neutral alumina and 5% EtOAc in hexane as eluent).

Step 3

A solution of 4-aryl-1-trityl-pyrazole (2.03mmol) in ether HCl (15 mL) was stirred for 1hr. The reaction mixture was then concentrated under reduced pressure and washed with hexane to afford 4-phenyl-*1H*-pyrazole hydrochloride as white solid. The residue was dissolved in ethyl acetate then washed with saturated NaHCO_3_, dried over sodium sulfate and concentrated to in vacuo to afford 4-aryl-1-H-pyrazole.

**4-phenyl-1H-pyrazole (2b)**

^1^H NMR (400 MHz, DMSO-*d*_6_) δ 13.01 (br, NH, 1H), 8.22 (s, 1H), 8.00 (s, 1H), 7.66 (d, J = 7.2 Hz, 2H), 7.40 (t, J = 7.7 Hz, 2H), 7.23 (t, J = 7.4 Hz, 1H) ppm. ^13^C NMR (101 MHz, DMSO-*d*_6_) δ 133.35, 129.23, 126.31, 125.56, 121.62, 66.81 ppm. (Figure S24)

**4-(4-(trifluoromethyl)phenyl)-1H-pyrazole (2c)**

^1^H NMR (400 MHz, DMSO-*d*_6_) δ 13.12 (br, NH, 1H), 8.36 (s, 1H), 8.05 (s, 1H), 7.83 (d, J = 8.2 Hz, 2H), 7.69 (d, J = 8.2 Hz, 2H) ppm. ^13^C NMR (101 MHz, DMSO-*d*_6_) δ 137.62, 137.15, 126.16, 126.12, 126.09, 126.05, 125.87, 120.31 ppm. ^19^F NMR (377 MHz, DMSO-*d*_6_) δ -60.76 ppm. m/z HRMS(ESI) found [M+H]^+^ 213.0650, C_10_H_8_F_3_N_2_^+^ requires 213.0634. (Figure S25)

**4-(4-(tert-butyl)phenyl)-1H-pyrazole (2d)**

^1^H NMR (400 MHz, DMSO-*d*_6_) δ 12.86 (s, 1H), 7.99 (s, 2H), 7.50 (d, *J* = 8.3 Hz, 2H), 7.35 (d, *J* = 8.3 Hz, 2H), 1.27 (s, 9H) ppm. ^13^C NMR (101 MHz, DMSO-*d*_6_) δ 148.66, 130.47, 125.91, 125.34, 121.51, 34.58, 31.59 ppm. (Figure S26)

**1-(4-(1H-pyrazol-4-yl)phenyl)ethanone (2f)**

^1^H NMR (400 MHz, DMSO-*d*_6_) δ 13.12 (br, NH, 1H), 8.36 (s, 1H), 8.06 (s, 1H), 7.93 (d, J = 8.3 Hz, 2H), 7.76 (d, J = 8.3 Hz, 2H), 2.56 (s, 3H) ppm. ^13^C NMR (101 MHz, DMSO-*d*_6_) δ 197.71, 138.24, 134.65, 129.46, 125.36, 120.71, 27.04 ppm. (Figure S28)

**Synthesis of ethynyl-1H-pyrazole (2e)**

Step 1

To a solution of 4-iodo-1H-pyrazole (1.93 g, 10 mmol) and ethynyl(trimethyl)silane (4.00 g, 40 mmol) in diethylamine (10 mL) were added bis(triphenylphosphine)- palladium(II) dichloride (350 mg, 0.5 mmol), and copper(I) iodide (44.5 mg, 0.25 mmol), and the reaction mixture was stirred at room temperature for 18 h. The solvent was removed in vacuo and the resulting residue was dissolved in Et_2_O (50 mL) and filtered. The filtrate was concentrated and the residue was purified by silica gel chromatography (SiO_2_, hexane:ethyl acetate mixtures). 4-((trimethylsilyl)ethynyl)-*1H*-pyrazole (0.88 g, 54 %) as a brown oil.

Step 2

To a solution of 4-((trimethylsilyl)ethynyl)-1H-pyrazole (0.82 g, 5 mmol) in MeOH (10 mL) was added a solution of potassium carbonate (1.38 g, 10 mmol) in water (10 mL). After stirring for 18 h at room temperature, the reaction mixture was neutralized with 2 N HCl and concentrated, the resulting residue was dissolved in Et_2_O (50 mL). The organic layer was washed with saturated brine solution, dried over sodium sulfate and concentrated to in vacuo. Purification by column chromatography (SiO_2_, hexane:ethyl acetate mixtures ). ^1^H NMR (400 MHz, Chloroform-*d*) δ 7.76 (s, 2H), 3.05 (s, 1H) ppm. ^13^C NMR (101 MHz, Chloroform-*d*) δ 137.32, 101.97, 78.59, 74.94 ppm. (Figure S27)

**5. Synthesis of biotin-azide**

Biotin-azide was generally prepared according to literature procedures with two steps.

Step 1

2-bromoethylamine hydrobromide (500 mg, 2.44 mmol) was add to a solution of sodium azide (475.9 mg, 7.32 mmol, 3 equiv.) in H_2_O (2 mL) at 75 °C for 21 h. After the reaction complete, cool the reaction mixture to 0 °C. Et_2_O (2 mL) and solid KOH (800 mg) was then added to the reaction mixture. Separate the organic phase and extract the aqueous layer with Et_2_O (3X10 mL). The combined organic layer was dried with MgSO_4_. The resulting solution was filtered and evaporated carefully by rotary evaporation (35 °C, 750 mbar) to afford 2-azidoethylamine.

Step 2

A solution of biotin (0.30 g, 1.23 mmol) in SOCl_2_ (5 mL) was kept at room temperature for 1 h. The reaction mixture was evaporated under vacuum and co-evaporated with anhydrous toluene (3 × 15 mL) to produce biotin acid chloride. The crude product was then dissolved in anhydrous acetonitrile (15 mL) and drop-wise added to a solution of 2-azidoethanamine (0.22 g, 2.56 mmol) and Et_3_N (523 μL, 3.68 mmol) in acetonitrile (15 mL). The reaction mixture was kept at room temperature for 4 h. Evaporation of the solvent yielded a crude product that was purified by column chromatography (EtOAc–MeOH 5:1) producing biotin azide (0.35 g, 92%). ^1^H NMR (400 MHz, DMSO-*d_6_*) δ 8.06 (t, *J =* 5.4 Hz, 1H), 6.45 (s, 1H), 6.38 (s, 1H), 4.30 (m, 1H), 4.15 – 4.04 (m, 1H), 3.33 (t, *J* = 5.7 Hz, 2H), 3.22 (q*, J* = 5.6 Hz, 2H), 3.12 – 3.05 (m, 1H), 2.81 (m, 1H), 2.57 (d, *J* = 12.4 Hz, 1H), 2.07 (t, *J* = 7.4 Hz, 2H), 1.68 – 1.20 (m, 6H) ppm. ^13^C NMR (101 MHz, DMSO-d6) δ 172.93, 163.22, 61.49, 59.66, 55.89, 50.44, 38.61, 35.60, 28.67, 28.50, 25.62 ppm. (Figure S29)

**6. General procedure for bioconjugation of phenylalanine contained tripeptide Ac-FVN-OMe**

To a solution of **Ac-FVN-OMe** (0.2 mmol, 1 equiv., 86.8 mg) in CH_3_CN/H_2_O (1.5 mL/1.5 mL) was added pyrazole **2a or 2e** (0.3 mmol) in presence of photosensitizer TPT^+^BF_4_^-^ (0.04 mmol, 20 mol%, 15.8 mg) under air atmosphere and irradiated by 3W blue LEDs at 25 ˚C for 10 h. After completion of the reaction, the solution was analyzed by ESI-HRMS.

7. Synthesis of biotin tagged tripeptide Ac-F(Pyr-Biotin)VN-OMe by CuAAC reaction.

Pyrazole labeled tripeptide carrying alkyne handles at the concentration 3 mM in CH_3_CN/H_2_O (1.5 mL/1.5 mL) were labeled with the CuAAC reaction. The reactions were performed by addition of 40 mM CuSO_4_, 20 mM biotin-azide and 40 mM sodium ascorbate. The reactions were then stirred for 12 hour at room temperature.

8. Procedure for visible-light-induced pyrazole tagging conjugated phenylalanine-targeted biomolecules

a. Polypeptides

To a solution of polypeptide (0.2 mmol, 1 equiv.) in CH_3_CN/H_2_O (1.5 mL/1.5 mL) was added pyrazole **2a** (0.3 mmol) in presence of photosensitizer TPT^+^BF_4_^-^ (0.04 mmol, 20 mol%, 15.8 mg) under air atmosphere and irradiated by 3W blue LEDs at 25 ˚C for 10 h. After completion of the reaction, the solution was analyzed by MS/MS spectroscopy.

b. Insulin

The reaction was performed in co-solvent (CH3CN/H2O = 1:1) with 3 mM pig insulin (m/z = 5777 Da) and 10 μM pyrazole in presence of 10 mM TPT^+^BF_4_^-^ under the irradiation of blue LED at r.t.. The reaction mixtures with different reaction time were monitored by MALDI-TOF-MS, MS/MS, and HRMS spectroscopies.

**9. SAXS measurements**

SAXS data for the pyrazole-bound insulin solutions were conducted at the 23A SWAXS endstation of National Synchrotron Radiation Research Center (NSRRC), using a beam of 15.0 keV (wavelength λ= 0.8267 Å) and a sample-to-detector distance 2503 mm. SAXS data were collected on a pixel detector Dectirs-Pilatus 1M detector of an active area of 169 x 179 mm^2^ and a detector pixel solution of 172 μm. The scattering wavelength $\mathbf{q=4}{}^{\boldsymbol{-1}}\boldsymbol{sin}$, defined by the scattering θand ${}$, was calibrated with a standard sample of silver behenate. To minimize radiation damages, the 5-mm sample solution with thin (12 μm) kapton windows was gently rocked within an area of 1.5 x 1.5 mm^2^ to avoid prolonged spot exposure (ca. 0.5 mm in beam diameter) of the sample solution and measured at room temperature. SAXS data were subtracted with buffer scattering measured under an identical environment as that used for the pyrazole-bound insulin sample solutions; the data were then corrected for incoming flux, sample thickness and electronic noise of the detector, as detailed in a previous report. (*7*)

**Supplementary Figures and Data**


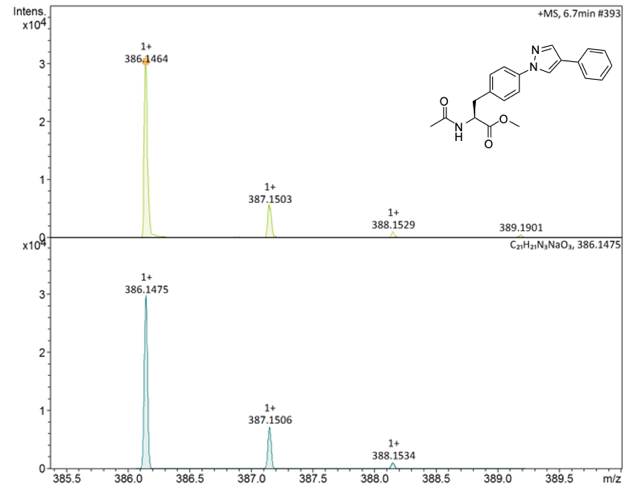
A B


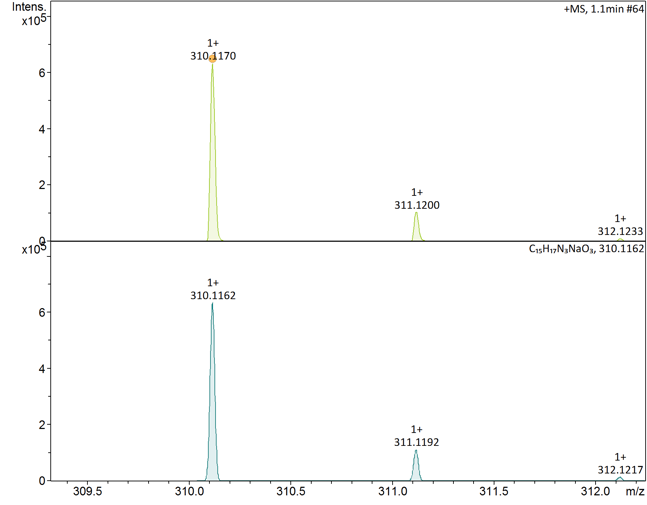


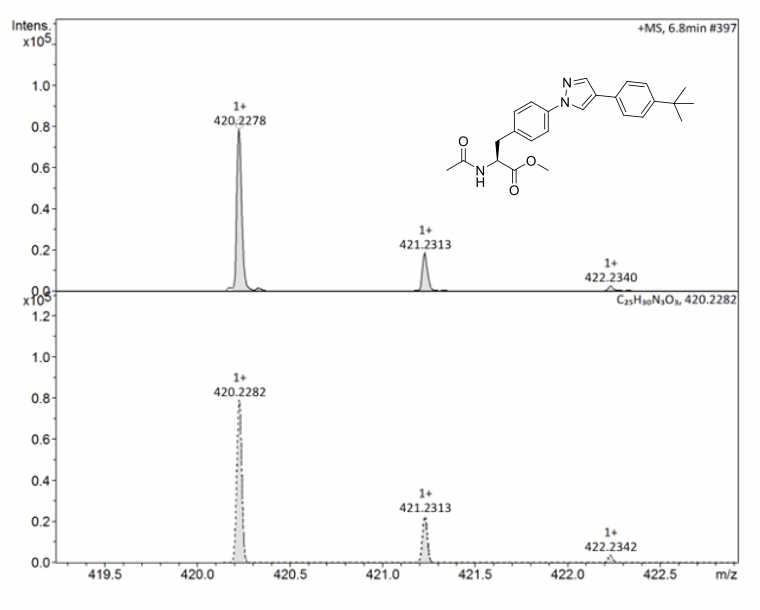
**C D**


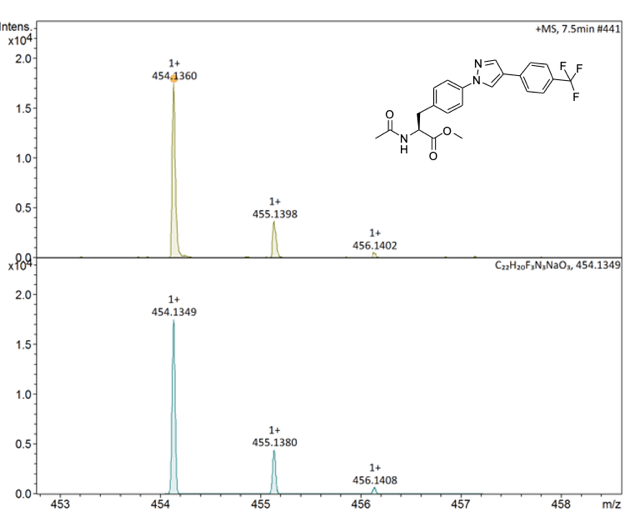


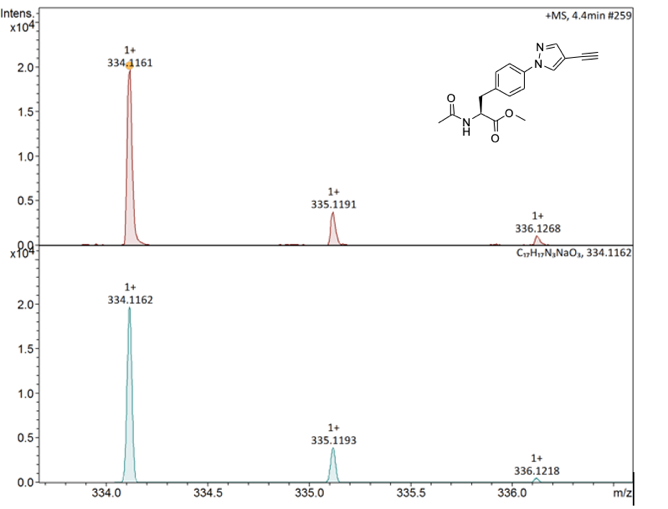

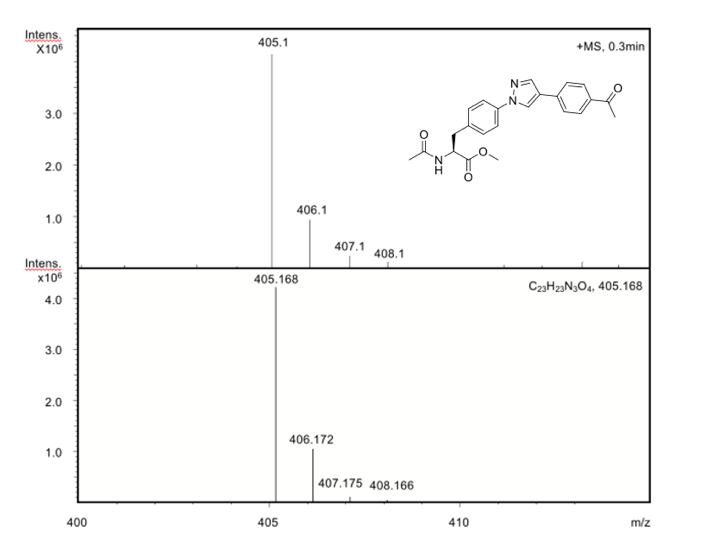


**E F**

**Fig S1. LC-MS analysis of amino acid modifications.**


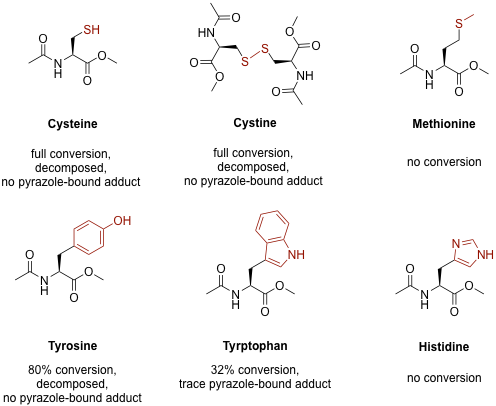


Fig. S2 Visible-light-induced reactivity of pyrazole with other natural amino acids. The reactions were performed in co-solvent (CH3CN/H2O = 1:1) with 3 mM amino acids and 10 μM pyrazole in presence of 10 mM TPT^+^BF_4_^-^ under the irradiation of blue LED at r.t.. The reaction mixtures were monitored by FID-GC and ESI-MS, using biphenyl as the internal standard.

A B


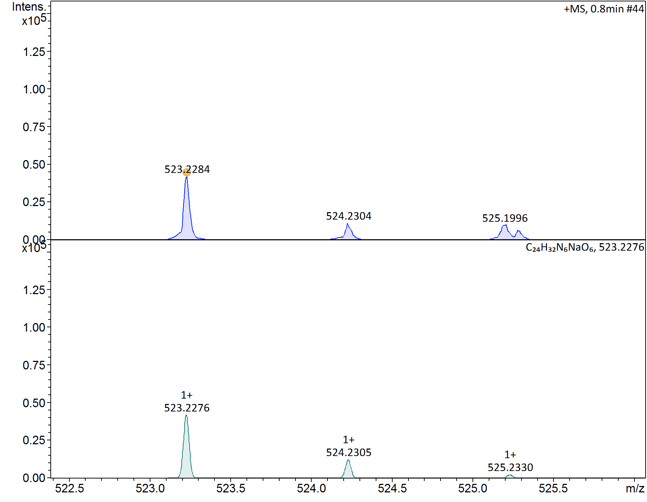

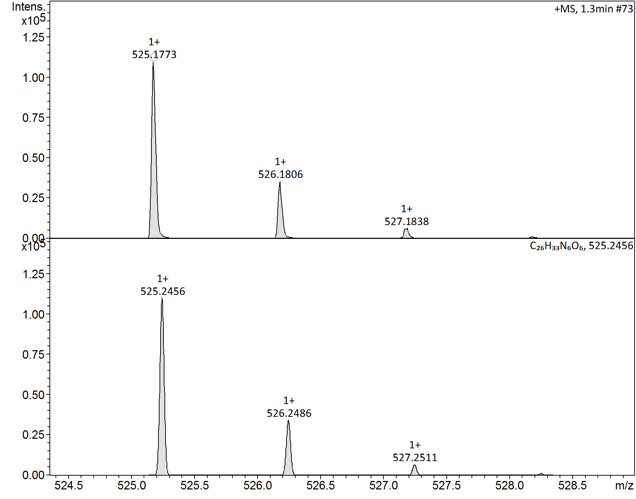


Fig S3. ESI-HRMS analysis of tripeptide modification. The modifications of tripeptide were analyzed by ESI-HRMS spectrum. (A) The ESI-HRMS spectrum of pyrazole-labelled tripeptide peaks at m/z = 523.2284, and the simulated fit for the C_24_H_32_N_6_NaO_6_ based on isotopic abundance at m/z = 523.2276. (B) The ESI-HRMS spectrum of 4-ethynyl-1H-pyrazole-labelled tripeptide peaks at m/z = 525.1773, and the simulated fit for the C_24_H_32_N_6_NaO_6_ based on isotopic abundance at m/z = 525.2456.

**
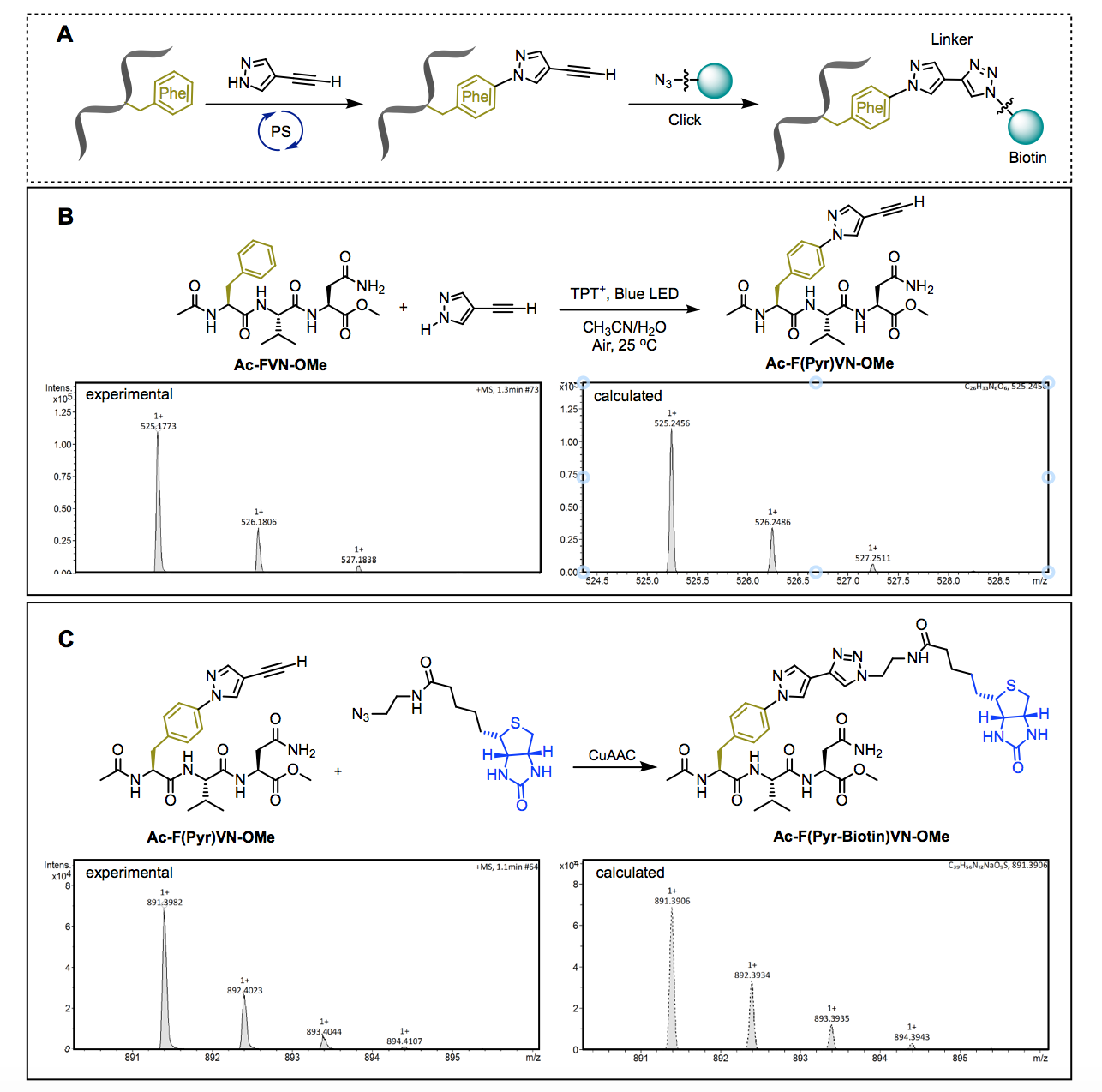
**

**Fig S4. ESI-HRMS analysis of the bio-orthogonal product of the tripeptide Ac-FVN-OMe** (**A**) Two-step process for functionalizing the phenylalanine-contained tripeptide (**Ac-FVN-OMe**). (**B**) Visible-light-induced bioconjugation of **Ac-FVN-OMe** (0.02 mmol) with 4-ethynyl-1*H*-pyrazole (0.3 mmol). The reaction conditions are shown with molecular weight listed for the corresponding conjugation. (**C**) Synthesis of the biotin tagged tripeptide **Ac-F(Pyr-Biotin)VN-OMe** by CuAAC reaction. The ESI-HRMS spectrum of ethynylpyrazole-bound tripeptide peaks (**Ac-F(Pyr)VN-OMe**) at *m/z* = 525.1773, and the simulated fit for the **Ac-F(Pyr)VN-OMe** based on isotopic abundance at m/z = 525.2456. For the Click reaction product **Ac-F(Pyr-Biotin)VN-OMe**, ESI-HRMS spectrum exhibiting a base peak at *m/z* = 891.3982, and its simulated peak of (**Ac-F(Pyr-Biotin)VN-OMe·CH_3_OH·Na^+^**) at *m/z* = 891.3906.

**
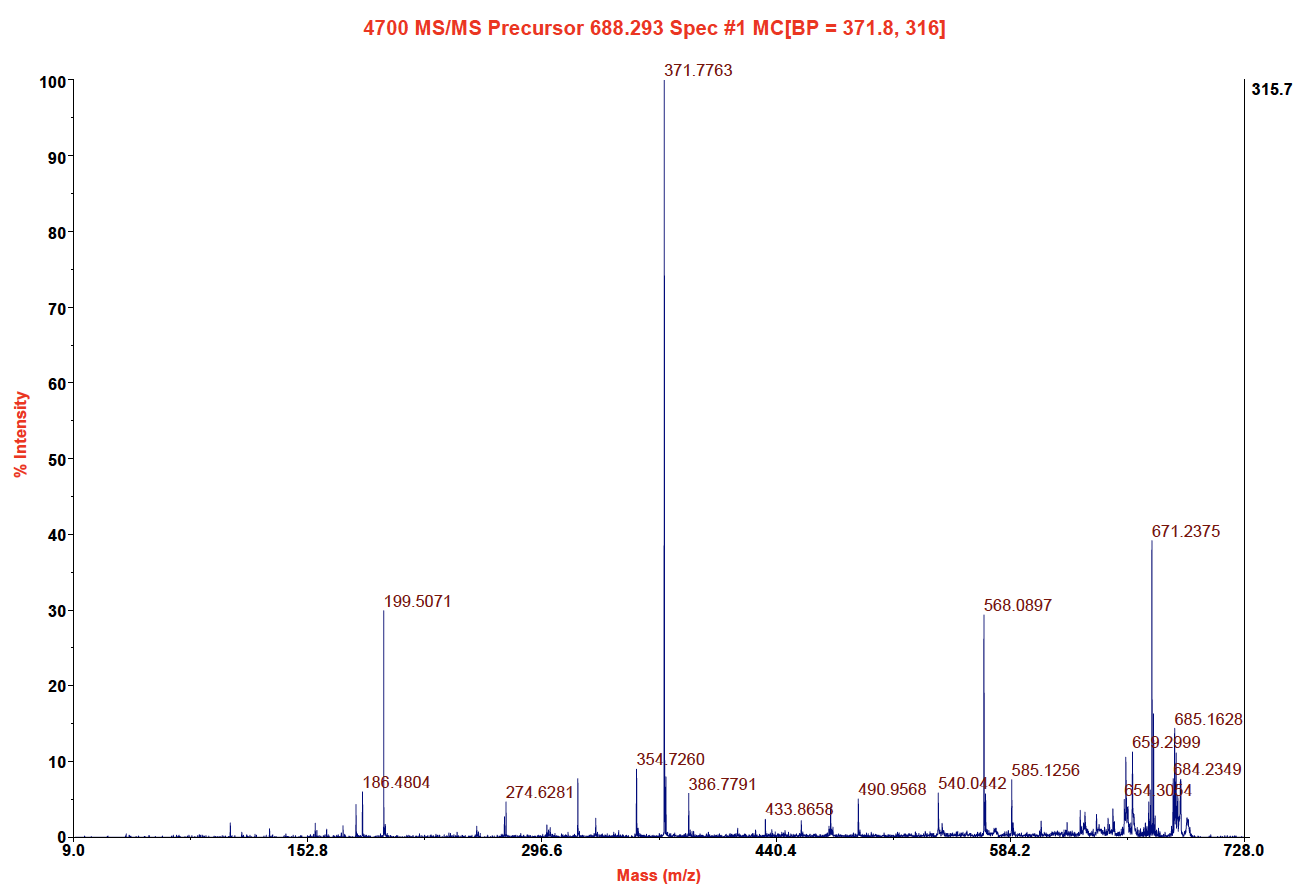
**

Fig S5. MALDI-TOF-MS/MS analysis of Penta-peptide RGDFS modification. The modification of penta-peptide was analyzed by MS/MS spectrum. MS/MS was key in confirming the site of pyrazolation. Predicted b and y ions are presented in the figure.

**OVA Peptide 257-264**

**
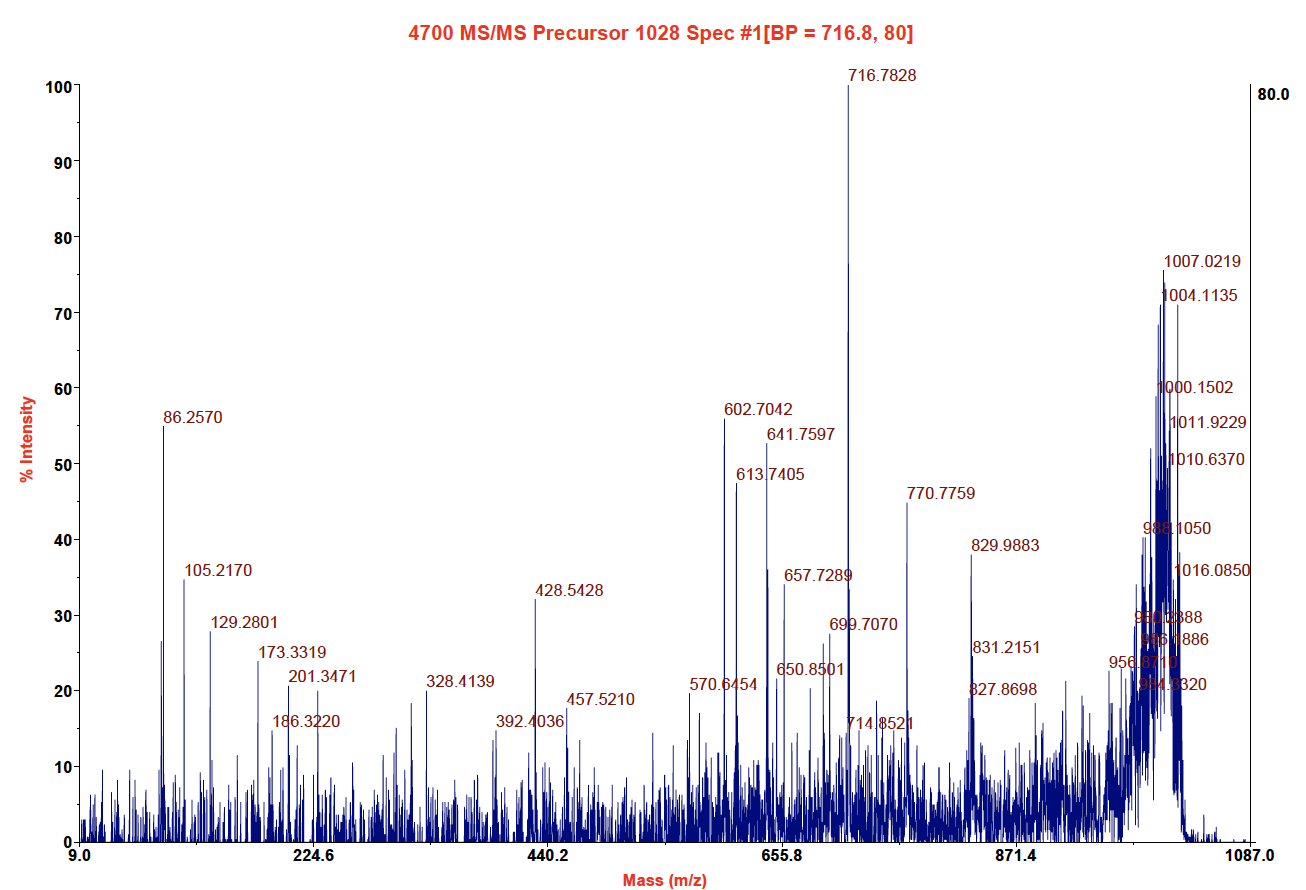
**

Fig S6. MALDI-TOF-MS/MS analysis of OVA Peptide 257-264 modification. The modification of polypeptide was analyzed by MS/MS spectrum. MS/MS was key in confirming the site of pyrazolation. Predicted b and y ions are presented in the figure. Observed b and y ions are highlighted in bold.

**Neuromedin B**

**
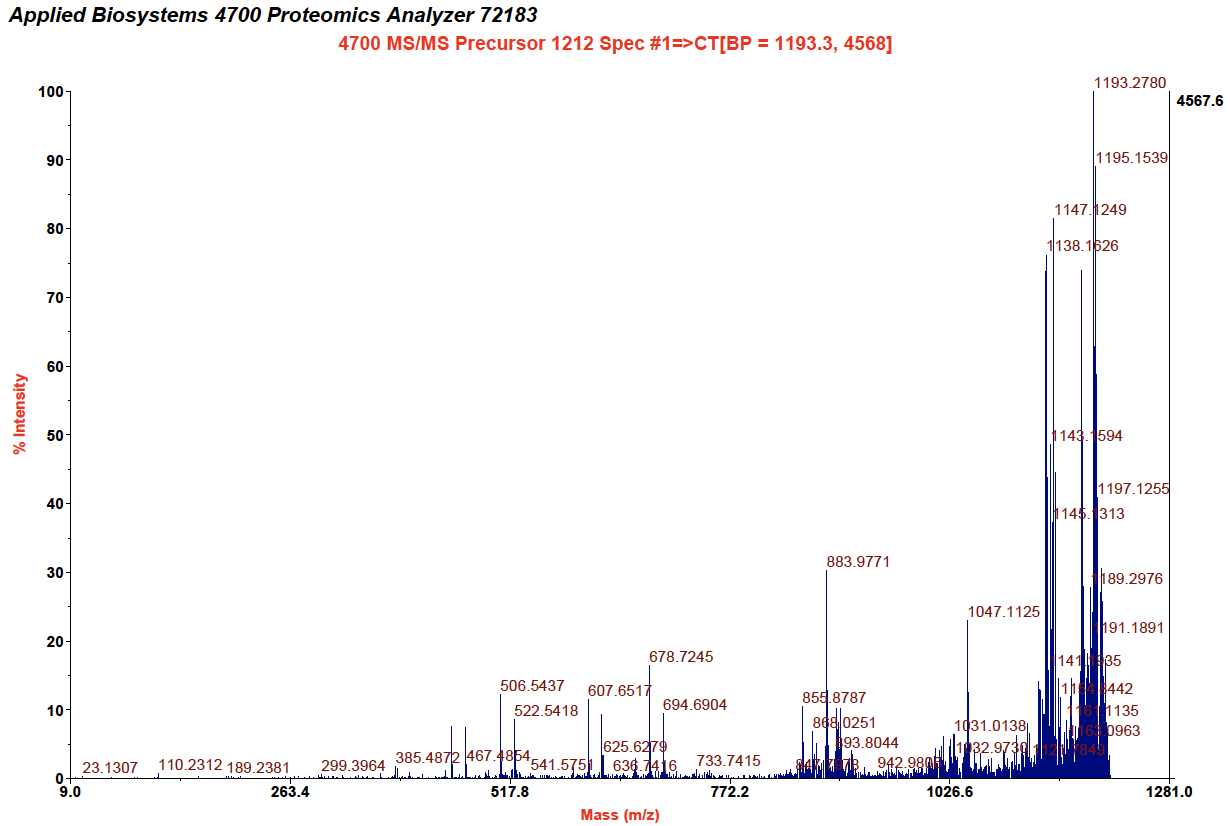
**

Fig S7. MALDI-TOF-MS/MS analysis of peptide Neuromedin B modification. The modification of polypeptide was analyzed by MS/MS spectrum. MS/MS was key in confirming the site of pyrazolation. Predicted b and y ions are presented in the figure. Observed b and y ions are highlighted in bold.

**Kisspeptin-10**

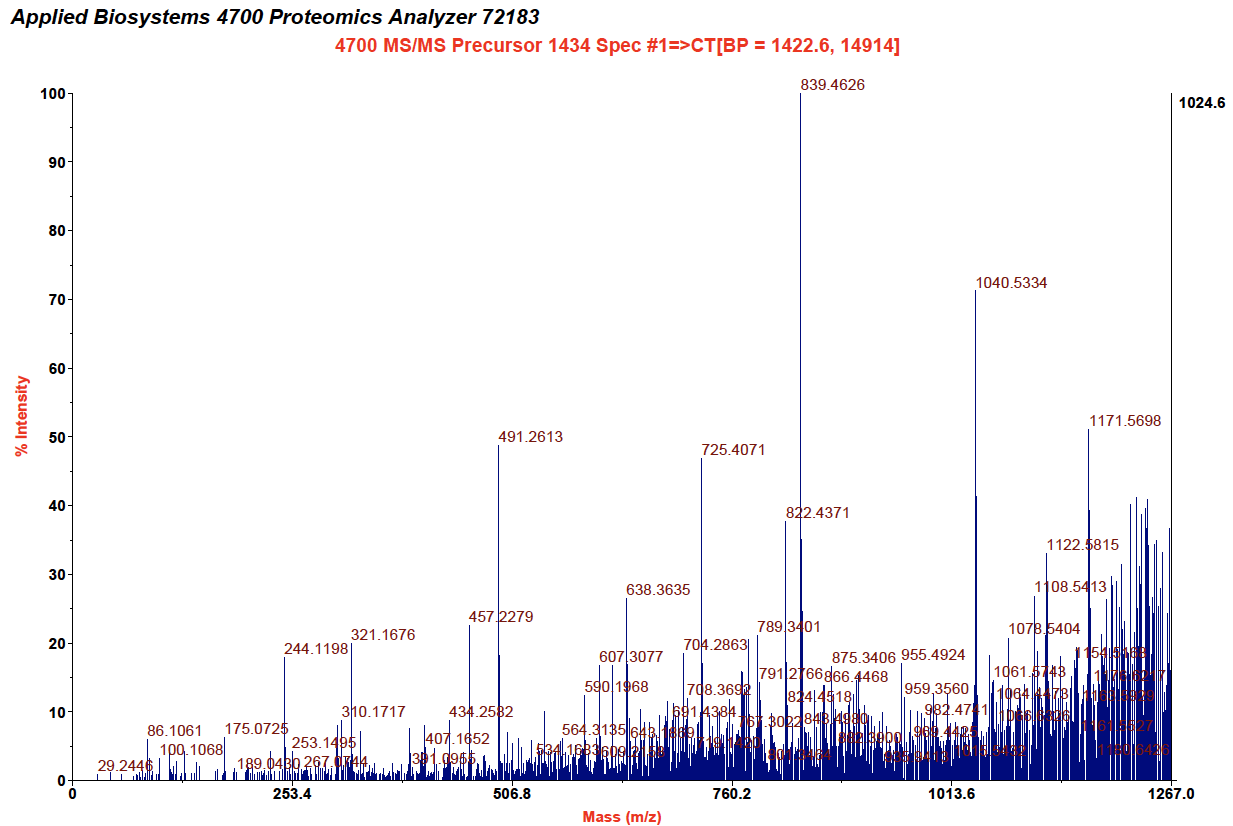


Fig S8. MALDI-TOF-MS/MS analysis of peptide Kisspeptin-10 modification. The modification of polypeptide was analyzed by MS/MS spectrum. MS/MS was key in confirming the site of pyrazolation. Predicted b and y ions are presented in the figure. Observed b and y ions are highlighted in bold.

**MOG 35-55**

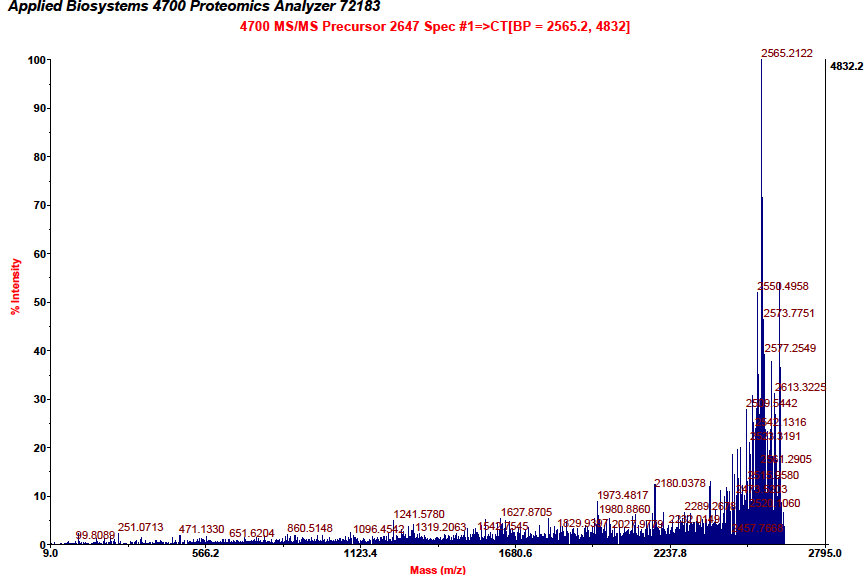


Fig S9. MALDI-TOF-MS/MS analysis of peptide MOG 35-55 modification. The modification of polypeptide was analyzed by MS/MS spectrum. MS/MS was key in confirming the site of pyrazolation. Predicted b and y ions are presented in the figure. Observed b and y ions are highlighted in bold.

**Insulin**

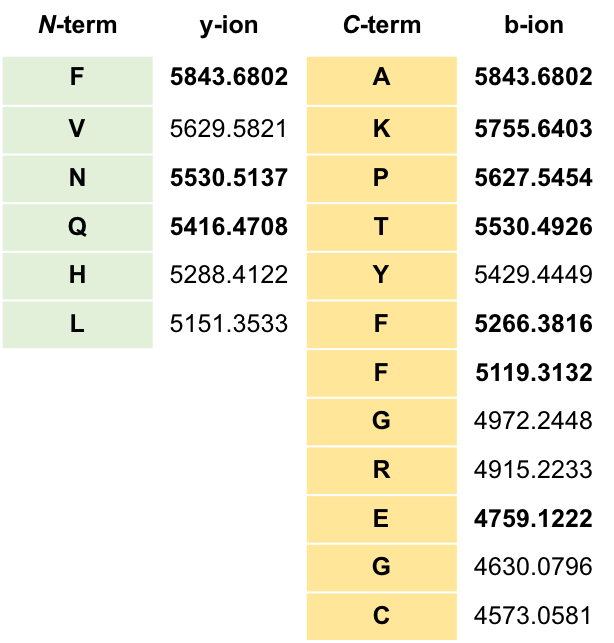


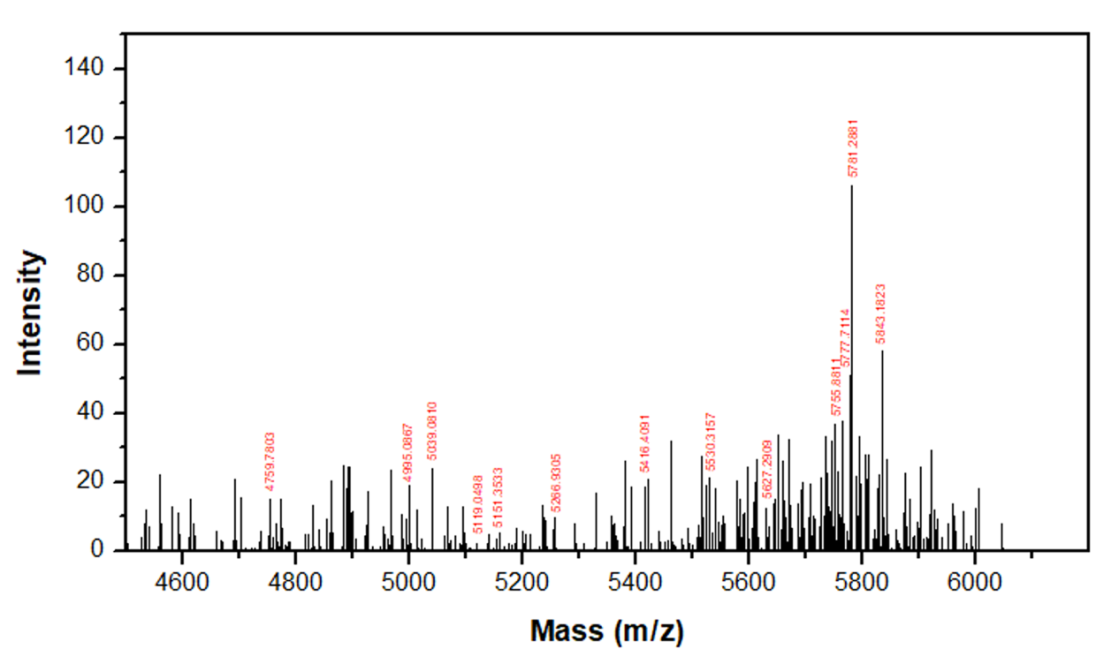


Fig S10. MALDI-TOF-MS/MS analysis of protein insulin modification. The modification of insulin was analyzed by MS/MS spectrum. MS/MS was key in confirming the site of pyrazolation. Predicted b and y ions are tabulated in the table. Observed b and y ions are highlighted in bold.


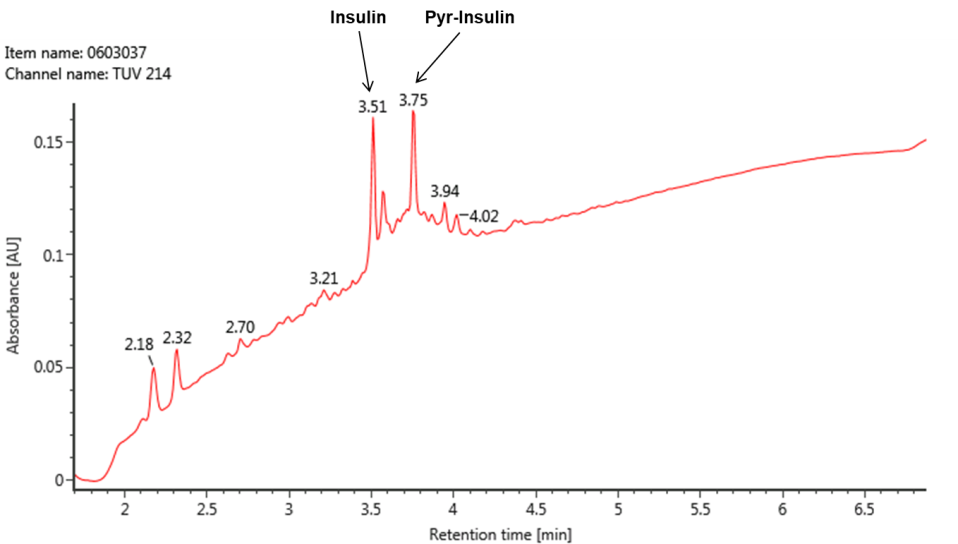


(A) (B)


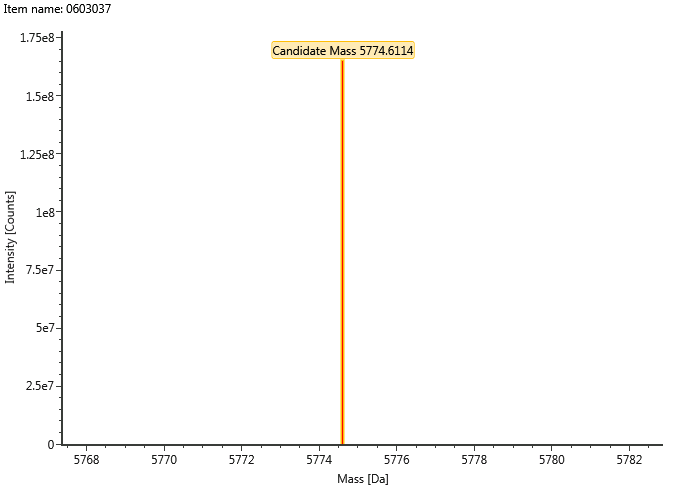

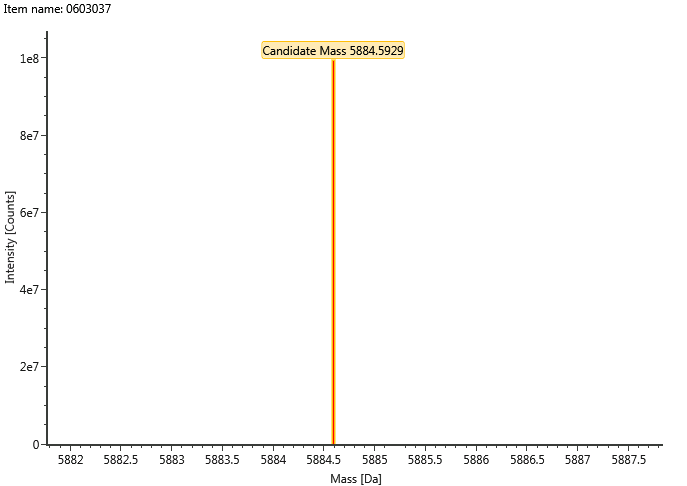


Insulin

Expected MS: 5774.6427

Found MS: 5774.6114

[Pyr-Insulin]CH_3_CN

Expected MS: 5884.7067

Found MS: 5884.5929

**Fig S11.** **LC-MS analysis of insulin and modified insulin.** (Top) LC spectrum of the insulin and pyrazole-bound insulin. (Bottom) LC-MS analysis of insulin without (A) and with (B) a pyrazole labeling. The expected mass and found mass are shown on the top right of deconvoluted peak.


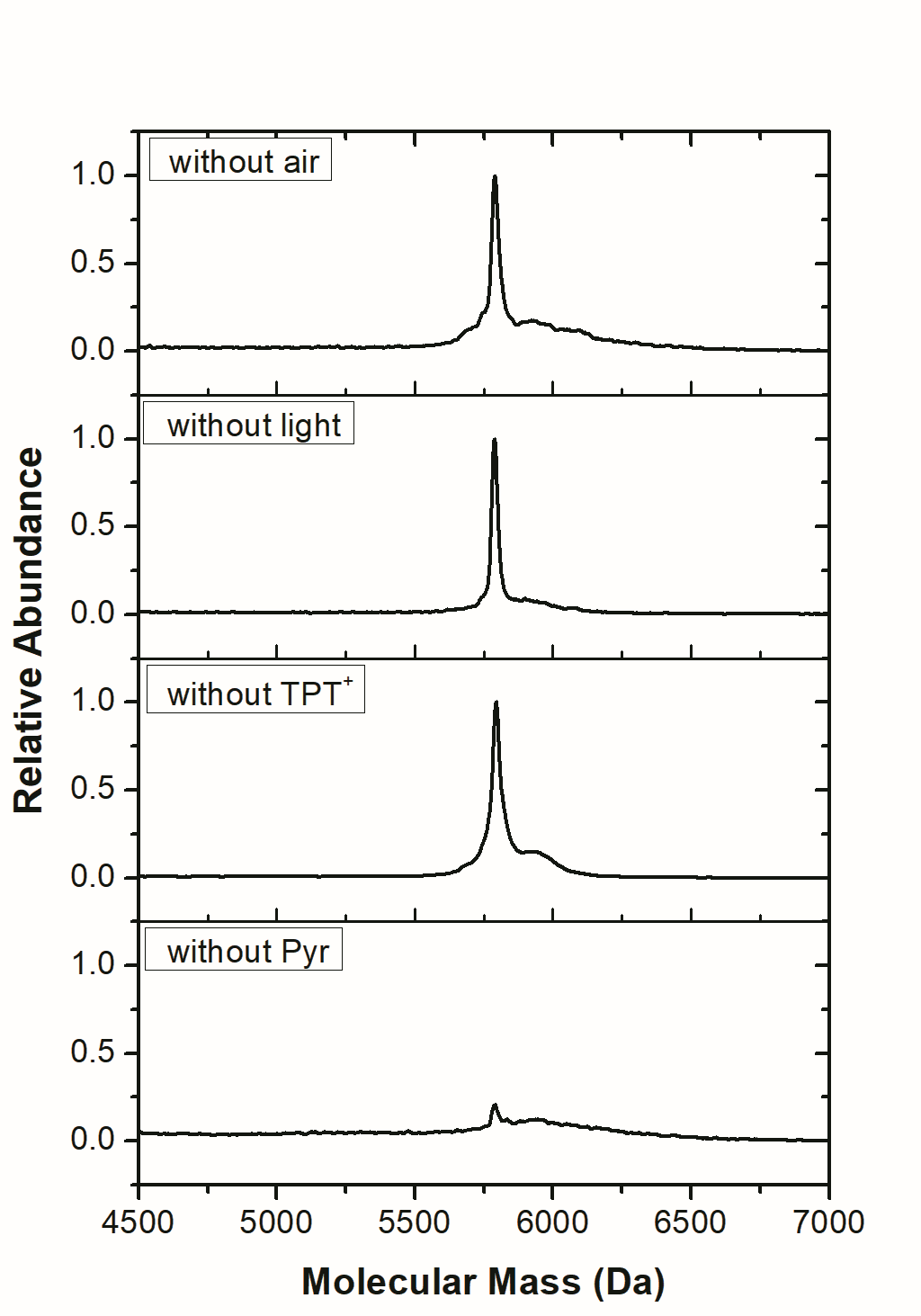


Fig. S12 Control experiments of visible-light-induced pyrazole tagging on insulin. The standard reaction was performed in co-solvent (CH3CN/H2O = 1:1) with 3 mM insulin and 10 μM pyrazole in presence of 10 mM TPT^+^BF_4_^-^ under the irradiation of blue LED at r.t.. The reaction mixtures were monitored by MALDI-TOF-MS.

**Fig. S13 X-Ray crystal data for pyrazole-phenylalanine adduct 3a**

CCDC 1912810 contain the supplementary crystallographic data for this paper.


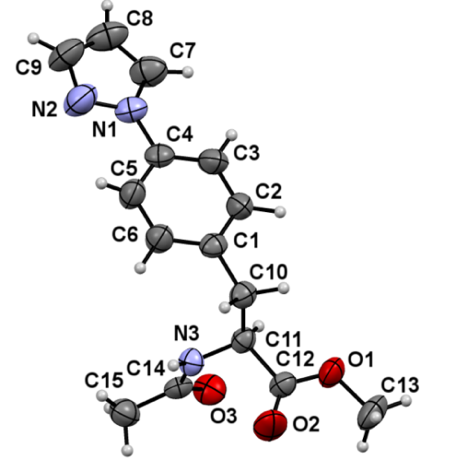


Crystal data and structure refinement for

Identification code 170423

Empirical formula C15 H17 N3 O3

Formula weight 287.32

Temperature 296 K

Wavelength 1.54178 Å

Crystal system Orthorhombic

Space group P2_1_2_1_2_1_

Unit cell dimensions a = 5.00990(10) Å α= 90°.

b = 8.4438(2) Å β= 90°.

c = 34.9640(7) Å γ = 90°.

Volume 1479.07(5) Å^3^

Z 4

Density (calculated) 1.290 Mg/m3

Absorption coefficient 0.755 mm^-1^

F(000) 608.0

Crystal size 0.12 x 0.14 x 0.16 mm^3^

Theta range for data collection 5.06 to 64.98°.

Index ranges -5<=h<=5, -9<=k<=9, -27<=l<=41

Reflections collected 11204

Independent reflections 2451 [R_int_ = 0.0495, R_sigma_ = 0.077]

Completeness to theta = 64.97° 99.0 %

Absorption correction multi-scan

Max. and min. transmission 0.7526 and 0.6179

Refinement method Full-matrix least-squares on F_2_

Data / restraints / parameters 11204/0/228

Goodness-of-fit on F2 1.158

Final R indices [I>2sigma(I)] R1 = 0.0558, wR2 = 0.1411

R indices (all data) R1 = 0.0566, wR2 = 0.1419

Largest diff. peak and hole 0.273/-0.300 e.Å-3


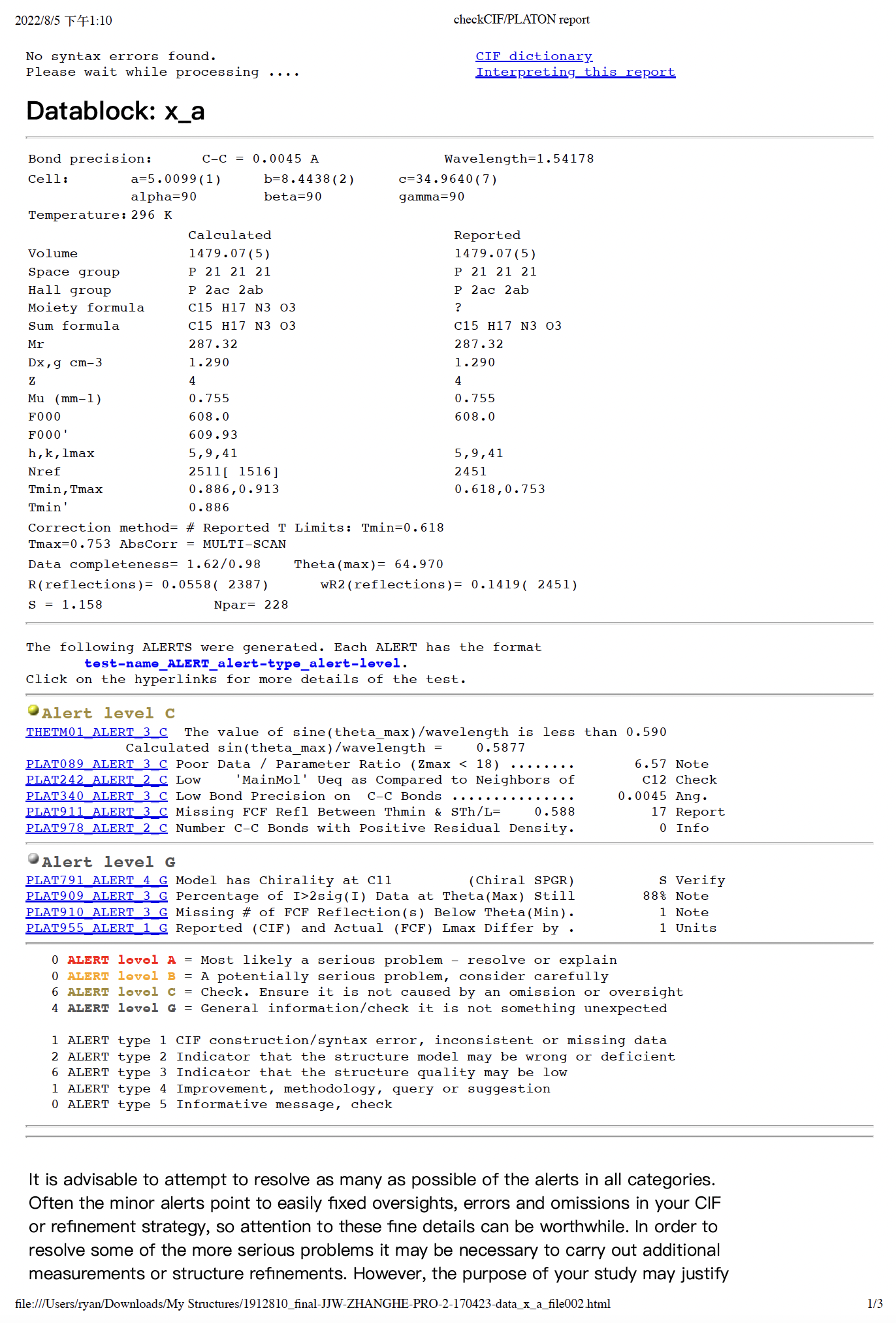


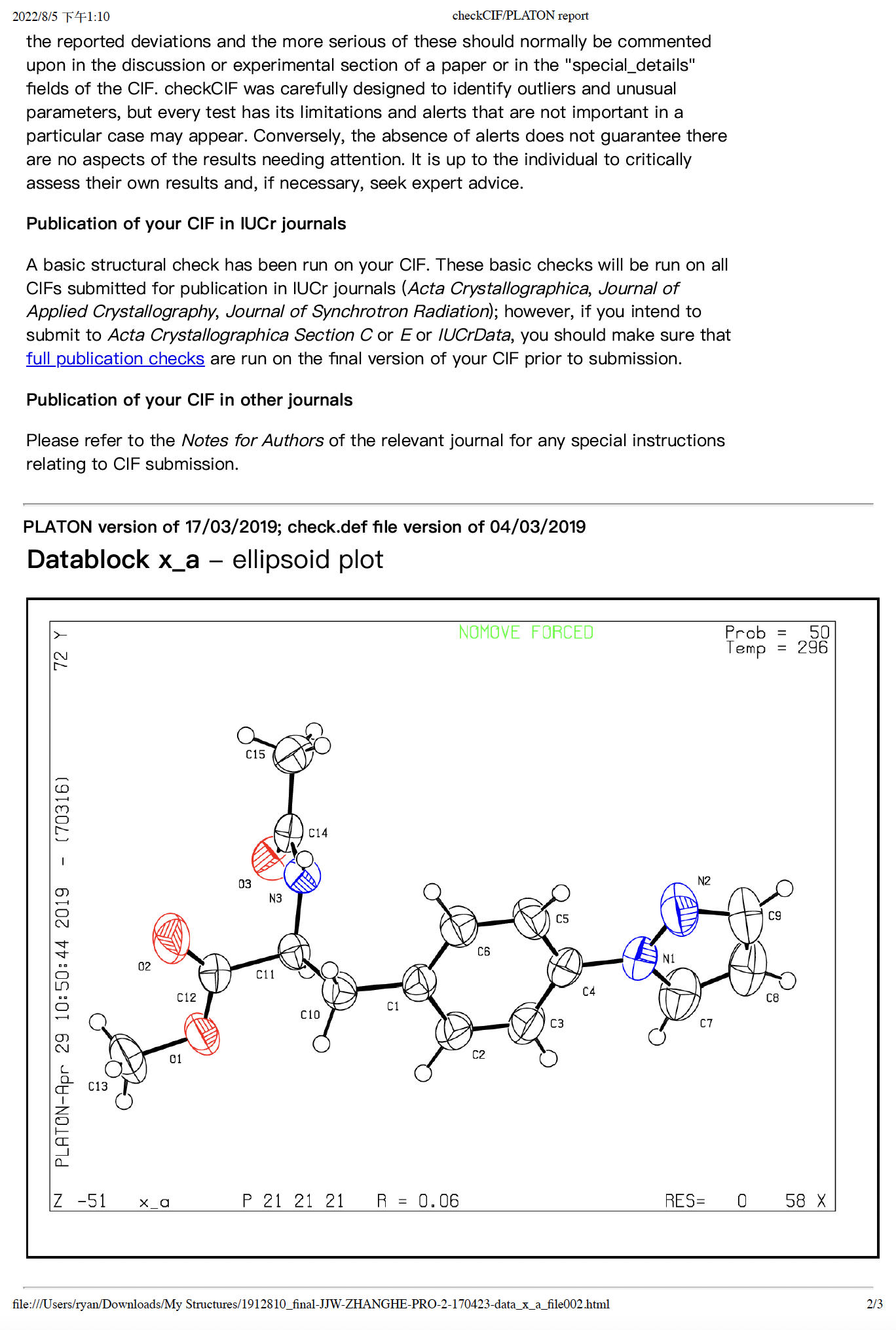


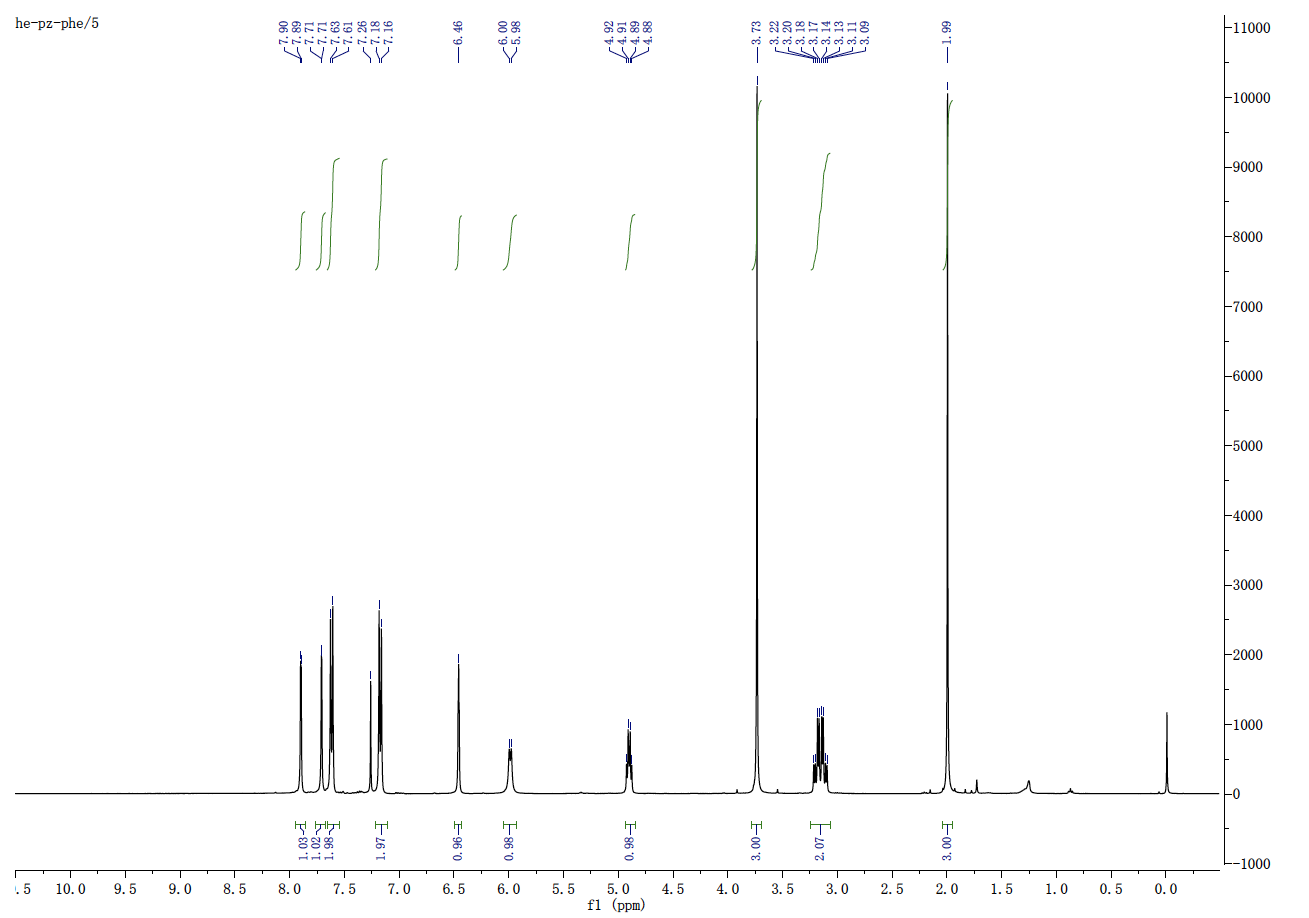

**Fig. S14 NMR data of 3a**

**Fig. S15 NMR data of biotin-azide**

**References**

1. E. D. Anderson, D. L. Boger, Inverse Electron Demand Diels–Alder Reactions of 1,2,3-Triazines: Pronounced Substituent Effects on Reactivity and Cycloaddition Scope. *J. Am. Chem. Soc.* **133**, 12285–12292 (2011).

2. E. J. Hanan *et al.*, Discovery of Selective and Noncovalent Diaminopyrimidine-Based Inhibitors of Epidermal Growth Factor Receptor Containing the T790M Resistance Mutation. *J. Med. Chem.* **57**, 10176–10191 (2014).

3. Y. Zhao *et al.*, Photocatalytic Cross-Dehydrogenative Amination Reactions between Phenols and Diarylamines. *ACS Catal.* **7**, 2446–2451 (2017).

4. E. Pedroso, A. Grandas, M. D. Ludevid, E. Giralt, Determination of the preferred tautomeric form of 4‐nitrohistidine. *J. Heterocyclic Chem*. **23**, 921–924 (1986).

5. R. A. Serwa, J.-M. Swiecicki, D. Homann, C. P. R. Hackenberger, Phosphoramidate-peptide synthesis by solution- and solid-phase Staudinger-phosphite reactions. *J. Pept. Sci.* **16**, 563–567 (2010).

6. I. A. Inverarity, R. F. H. Viguier, P. Cohen, A. N. Hulme, Biotinylated Anisomycin:  A Comparison of Classical and “Click” Chemistry Approaches. *Bioconj. Chem.* **18**, 1593–1603 (2007).

7. U.-S. Jeng *et al.*, A small/wide-angle X-ray scattering instrument for structural characterization of air–liquid interfaces, thin films and bulk specimens. *J. Appl. Cryst*. **43**, 110–121 (2009).
